# Design of Fungal Co-Cultivation Based on Comparative Metabolomics and Bioactivity for Discovery of Marine Fungal Agrochemicals

**DOI:** 10.3390/md18020073

**Published:** 2020-01-23

**Authors:** Ernest Oppong-Danquah, Paulina Budnicka, Martina Blümel, Deniz Tasdemir

**Affiliations:** 1 GEOMAR Centre for Marine Biotechnology (GEOMAR-Biotech), Research Unit Marine Natural Products Chemistry, GEOMAR Helmholtz Centre for Ocean Research Kiel, Am Kiel-Kanal 44, 24106 Kiel, Germany; eoppong-danquah@geomar.de (E.O.-D.); pbudnicka@gmail.com (P.B.); mbluemel@geomar.de (M.B.); 2 Faculty of Mathematics and Natural Science, Kiel University, Christian-Albrechts-Platz 4, 24118 Kiel, Germany

**Keywords:** *Plenodomus*, *Pyrenochaeta*, co-culture, metabolomics, sediment-derived fungi, phytopathogen, molecular network

## Abstract

Microbial co-cultivation is employed for awakening silent biosynthetic gene clusters (BGCs) to enhance chemical diversity. However, the selection of appropriate partners for co-cultivation remains a challenge. Furthermore, competitive interactions involving the suppression of BGCs or upregulation of known, functional metabolite(s) during co-cultivation efforts is also common. Herein, we performed an alternative approach for targeted selection of the best co-cultivation pair. Eight marine sediment-derived fungi were classified as strong or weak, based on their anti-phytopathogenic potency. The fungi were co-cultured systematically and analyzed for their chemical profiles and anti-phytopathogenic activity. Based on enhanced bioactivity and a significantly different metabolite profile including the appearance of a co-culture specific cluster, the co-culture of *Plenodomus influorescens* (strong) and *Pyrenochaeta nobilis* (weak) was prioritized for chemical investigation. Large-scale co-cultivation resulted in isolation of five polyketide type compounds: two 12-membered macrolides, dendrodolide E (**1**) and its new analog dendrodolide N (**2**), as well as two rare azaphilones spiciferinone (**3**) and its new analog 8a-hydroxy-spiciferinone (**4**). A well-known *bis*-naphtho-γ-pyrone type mycotoxin, cephalochromin (**5**), whose production was specifically enhanced in the co-culture, was also isolated. Chemical structures of compounds **1**–**5** were elucidated by NMR, HRMS and [α]${}_{D}^{20}$ analyses. Compound **5** showed the strongest anti-phytopathogenic activity against *Xanthomonas campestris* and *Phytophthora infestans* with IC_50_ values of 0.9 and 1.7 µg/mL, respectively.

## 1. Introduction

Plant diseases caused by bacterial and fungal pathogens pose severe losses to the global agroeconomy by directly reducing crop yield and quality [1]. The control of plant diseases is mainly achieved by spraying with conventional pesticides, although there is ample evidence of negative impacts on human health and the environment [2,3]. The development of resistance, as well as legal restrictions to conventional pesticides necessitate the search for natural, alternative plant protective agents [4]. Towards this aim, fungi and their secondary metabolites have a long-standing history. Their applicability for plant disease control involves direct introduction of the fungus into the soil as a biocontrol agent, as exemplified with *Trichoderma* sp. [5], and use of their secondary metabolites in direct application or as lead compounds for the synthesis of new chemical pesticides. Strobilurin type fungal metabolites are currently among the most important fungicides with about 25% of the market share [6]. The success of strobilurin, a polyketide originally isolated from the terrestrial fungus *Strobilurius tenacellus* in the 1970s, prompted exploration of the terrestrial fungi for new agrochemicals. Recently, fungi from marine environments have also been attracting research interest due to their capacity to produce unique bioactive metabolites as a consequence of environmental adaptation [7]. The increasing number of sequenced marine fungal genomes reveals large proportions (up to 10%–15%) of secondary metabolite biosynthetic gene clusters (BGC). However, the majority of these BGCs remain silent under standard laboratory culture conditions [8,9,10]. Co-cultivation approach is hence emerging as a powerful tool for activation of dormant BGCs [11] to unlock the hidden potential of marine fungi.

Microorganisms form complex multispecies communities in all environments. Over evolutionary time scales, they have acquired genetic modifications, which have led to their adaptation and specialization [12]. This has resulted in diverse microbial interactions, which may be positive, such as mutualism and commensalism, or negative such as competition, antagonism, and parasitism [13]. Previous co-cultivation studies have provided evidence for the induction and suppression of biosynthetic pathways. For example, the biosynthesis of diterpenoids libertellenones A and D was induced in a fungus *Libertella* sp. by a marine α-proteobacterium CNJ-328 in a co-culture [14]. Another form of induction was observed during the co-culture of the fungus *Emericella* sp. with an actinomycete *Salinispora arenicola* that led to a 100-fold increase in production of emericellamides A and B [15]. In another study, the transcriptional responses of a fungus *Penicillium chrysogenum* during co-cultivation with *Bacillus subtilis* was investigated and both polyketide synthase (PKS) and nonribosomal peptides synthase (NRPS) genes were found to be suppressed in the *Penicillium* strain [16].

Four major types of microbial interactions have been visualized on solid substrates: (i) distance inhibition (growth stops at a distance between competing strains), (ii) zone line (a dark precipitate is observed between competing strains), (iii) contact inhibition (competing strains grow large enough to contact each other), and (iv) overgrowth (one strain overgrowing the other) [17]. This indicates that microbial interactions lead to complex regulatory mechanisms, which unlock, induce, and/or suppress the biosynthesis of diverse molecules necessary for competition and communication [18]. Co-cultivation therefore aims to simulate ecological interactions for drug discovery. The selection of suitable microorganism pairs for co-cultivation, however, still remains a challenge. Many successful co-cultivation experiments have been based on educated guesses, trial and error attempts, or serendipity [10]. Other studies have used a ‘model microbe’ approach, in which a well-characterized microorganism is co-cultivated with a variety of other microbes [19,20]. Serendipitous co-cultivation of fungi and bacteria from same or different environments has also led discovery of novel small molecule natural products [15,21], however this is a laborious and rather untargeted approach. Hence, more targeted approaches for selection of an informed co-cultivation pair are necessary.

Herein, we report on an alternative approach for targeted selection of fungal isolates from the same marine environmental niche for co-cultivation. Eight marine sediment-derived isolates previously isolated from Windebyer Noor (Baltic Sea) [21] and identified as *Plenodomus influorescens*, *Penicillium bialowiezense*, *Sarocladium strictum*, Helotiales sp., two *Pyrenochaeta* strains, and two *Lentithecium* strains were cultivated on a small-scale on solid potato dextrose agar medium (PDA), followed by extraction with EtOAc. The individual isolates were classified as “weak” or “strong” based on the potency of their anti-phytopathogenic activity against a selection of phytopathogens. A systematic co-cultivation including combinations of weak-weak, strong-strong and weak-strong isolates was then carried out. This was followed by analysis of mono- and co-cultures by comparative metabolomics (involving molecular networking (MN)) and anti-phytopathogenic activity. Based on enhanced bioactivity and a significantly different metabolite profile, the co-culture *P. influorescens* (strong partner) with *Pyrenochaeta nobilis* (weak partner) was prioritized for large-scale co-culture and downstream chemical analysis. Fractionation and MN-guided isolation yielded five polyketide type compounds, **1**–**5**. Herein, we outline the selection process of the most promising co-culture followed by isolation, structure elucidation, and anti-phytopathogenic activity assessments of its constituents, **1**–**5**.

## 2. Results

### 2.1. Classification of Sediment-Derived Fungi as “Weak” and ”Strong”

In a previous study [21], we isolated eight marine fungi from Baltic Sea-derived sediment (Table 1). In the current investigation, we cultivated the fungal isolates individually (in triplicate) in solid potato dextrose agar (PDA) medium for 21 days. Their EtOAc extracts were tested for inhibitory activity against a panel of six phytopathogens, including four bacteria (*Erwinia amylovora*, *Pseudomonas syringae*, *Ralstonia solanacearum*, *Xanthomonas campestris*), a fungus (*Magnaporthe oryzae*), and an oomycete (*Phytophthora infestans*). In vitro anti-phytopathogenic activity (tested at 100 µg/mL) served as decisive criterion for categorizing the strains as “strong” (threshold of > 80% inhibitory activities against at least two phytopathogenic strains) or “weak” (below the set threshold) partners. This threshold was adopted to ensure that only very potent extracts were considered to be strong. Three strains, namely Helotiales sp., *P. influorescens*, and *P. bialowiezense* inhibited the phytopathogens *M. oryzae* and *P. infestans* (89%–99% inhibition; Table 1). Helotiales sp. (identified to order level, Figure S1) exhibited the broadest activity by further inhibiting the growth of a third plant pathogen *X. campestris* (83% inhibition, Table 1). These three strains were therefore classified as strong candidates. The other five strains comprising two *Lentithecium* sp., *S. strictum* and two *Pyrenochaeta* sp. showed variable inhibitory activities (25%–89%) against one single phytopathogen *P. infestans*, and were thus classified as weak (Table 1).

**Figure 1:**
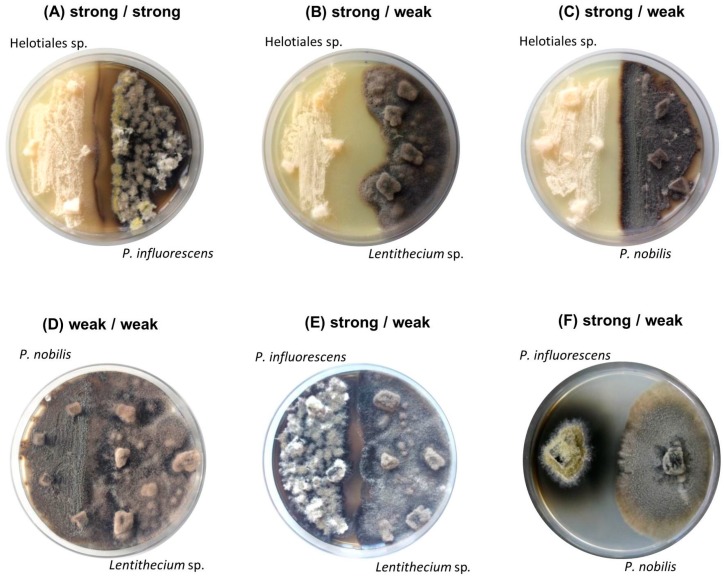
Images of six co-cultures after 21 days of incubation: (**A**–**C**) strong Helotiales (**left** side of the plate) (**A**) against strong *P. influorescens*; (**B**) against weak *Lentithecium* sp.; (**C**) against weak *P. nobilis* (**right**); (**D**) weak *P. nobilis* (**left**) against weak *Lentithecium* sp.; (**E**–**F**) strong *P. influorescens* (**left** side of the plate) (**E**) against weak *Lentithecium* sp.; (**F**) against weak *P. nobilis* (**right**).

The genus *Penicillium* is among the most fruitful and successfully studied fungal genera in terms of its ability to produce bioactive secondary metabolites [22]. With over 2200 documented isolated natural products reported in the Dictionary of Natural Products (DNP) [23] alone, new molecules are continually isolated from *Penicillium* [24]. Since the focus of our study was placed on less studied fungal genera, *P. bialowiezense* (strong) was excluded from further analyses. The yeast-like *S. strictum* (weak) was also excluded from further analysis due to having a very poor crude extract yield. The remaining four selected strains (two strong and two representative weak strains) were co-cultured on solid PDA medium at a small scale. This resulted in total six co-cultures, i.e. Helotiales sp. (strong)—*P. influorescens* (strong); *P. nobilis* (weak)—*Lentithecium* sp. (weak) and four combinations of one strong and one weak isolate: Helotiales sp. (strong)—*P. nobilis* (weak); Helotiales sp. (strong)—*Lentithecium* sp. (weak), *P. influorescens* (strong)—*P. nobilis* (weak) and *P. influorescens* (strong)*—Lentithecium* sp. (weak) (Figure 1, Table 1).

### 2.2. Selection of Co-Cultivation Pair for Large-Scale Co-Cultivation

Crude EtOAc extracts of the small-scale co-cultures (Figure 1) were examined for their bioactivity against our in-house panel comprising six phytopathogens. Furthermore, tandem mass spectrometry (MS/MS) based comparative metabolomics including molecular networking (MN) was performed for all co-culture pairs against their respective single cultures (Figures S2–S6).

Macroscopically, all co-cultures involving the strong candidate Helotiales sp. showed distinct growth areas and a pronounced mycelia-free zone between the competing strains, described as distance inhibition (Figure 1A–C). In contrast, a mutual type of interaction was observed between two weak candidates (*P. nobilis* - *Lentithecium* sp.) where fungal isolates displayed mycelial contact (Figure 1D). The co-cultures of the strong partner *P. influorescens* showed different interaction types. When *P. influorescens* (strong) was co-cultured with *Lentithecium* sp. (weak), contacts between both mycelia were observed at certain spots on the plate (Figure 1E), whereas the co-cultivation of *P. influorescens* with *P. nobilis* (weak) displayed a distance inhibition with a pronounced, dark-coloured zone of inhibition (Figure 1F).

**Figure 2:**
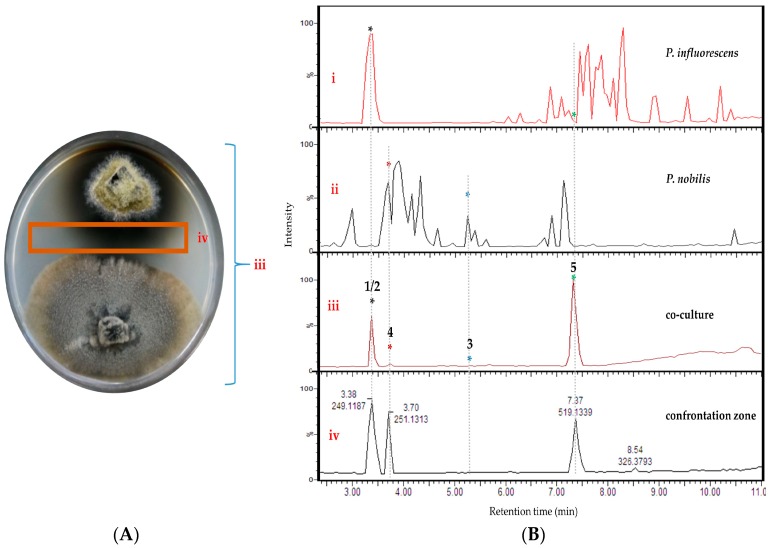
(**A**) Image of the co-culture of *P. influorescens* (strong partner, top strain) and *P. nobilis* (weak partner, bottom strain) after 21 days of incubation. (**B**) UPLC-MS base peak chromatograms of extracts deriving from (i) mono-culture of *P. influorescens*, (ii) mono-culture of *P. nobilis*, (iii) the whole co-culture, and (iv) confrontation zone.

**Figure 3:**
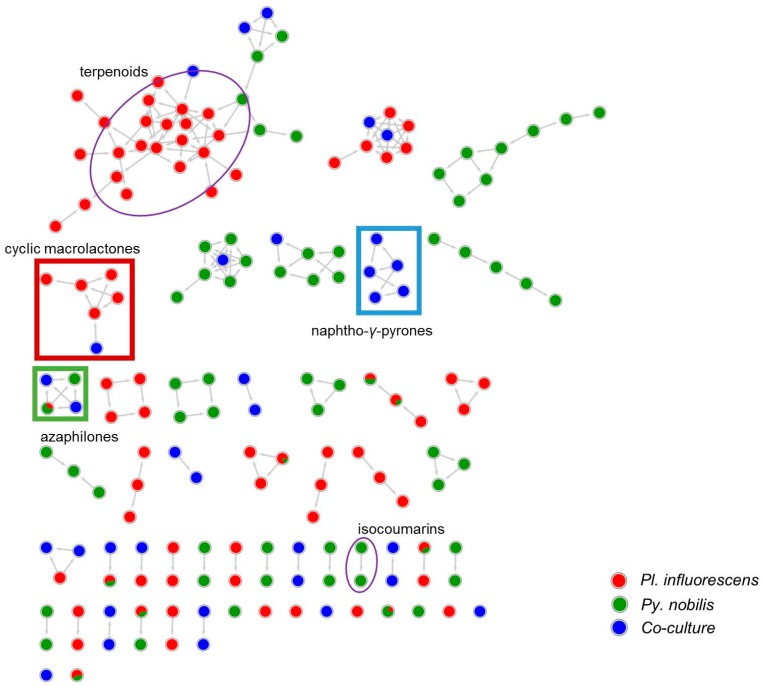
MN of the extracts of *P. influorescens* monoculture (red), *P. nobilis* monoculture (green) and their co-culture (blue). Cyclic macrolactone cluster is highlighted in red rectangle and naphtho-γ-pyrone cluster in blue rectangle. Azaphilone cluster is highlighted in green square. All other annotated clusters are highlighted in purple oval.

A detailed MN-based comparative metabolomics study showed that co-cultivation increased the size of several molecular families by inducing derivatives of compounds produced in the axenic mono-cultures (Figures S2–S6). However, no distinct co-culture specific major peaks were observed in the UPLC-MS chromatograms of most co-cultures (Figures S2–S6). As shown in Figure 2, only the co-culture of *P. influorescens* (strong) with *P. nobilis* (weak) coincided with the induction of a distinct major peak (**5**) that was also visualized in the MN (Figure 3). A molecular family cluster (MF) containing co-culture specific nodes was detected and putatively annotated as *bis*-naphtho-γ-pyrone (Figure 3). Other putatively annotated clusters in this MN included terpenoids, cyclic macrolactones, azaphilones, and isocoumarins (Figure 3). In total, 21 MFs (clusters of more than two nodes) were identified in the co-culture of *P. influorescens* (strong) with *P. nobilis* (weak).

Analysis of the UPLC-MS chromatograms of the *P. influorescens* and *P. nobilis* co-culture extracts showed unexpected pattern of distinctly suppressed peaks in the whole co-culture compared to mono-cultures. In the *P. influorescens* mono-culture (Figure 2B, i), the majority of the peaks appear at *t*_R_ 6.5–8.5 min, indicating the presence of mid- to non-polar compounds, whereas the *P. nobilis* mono-culture (Figure 2B, ii) contained most of the peaks at *t*_R_ 3–5 min, suggestive of rather polar compounds. In the whole co-culture (Figure 2A,B, iii) a newly induced peak at *t*_R_ 7.37 min (compound **5**) was observed, while almost all other peaks, except for the peak at *t*_R_ 3.38 min, of the respective mono-cultures were significantly suppressed. The organic extract of the confrontation zone alone (Figure 2A,B, iv) contained three major peaks at *t*_R_ 7.37 (**5**), 3.38 (**1** and **2**) and 3.70 (**4**). The latter (**4**) was present in the whole co-culture extract (Figure 2B, iii) in very low levels. The whole co-culture also contained compound **3**, which is the constituent of *P. nobilis*, in minute amounts (Figure 2B, iii and iv).

These observations were highly suggestive for a competitive interaction involving suppression of biosynthetic pathways of one strain, and upregulation of highly functional metabolite(s) mediating the competitive interaction by the other. The whole co-culture extract of *P. influorescens—P. nobilis* showed an enhanced activity (59%) against the phytopathogenic bacterium *X. campestris* (Table 1), which was further enhanced to 90% when the extract of the confrontation zone (iv, Figure 2B) was tested (Table 1). Hence, the whole co-culture *P. influorescens*—*P. nobilis* was prioritized for chemical work-up for targeted compound isolation.

### 2.3. MN-Guided Isolation and Structure Elucidation

**Figure 4:**
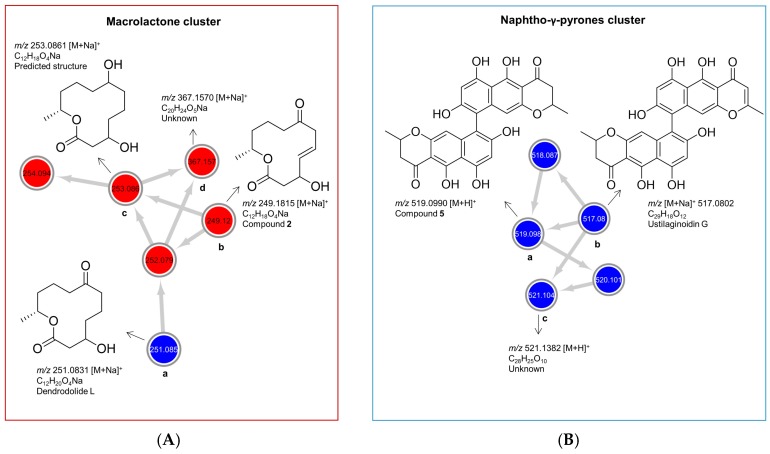
Molecular clusters of (**A**) cyclic macrolactones and (**B**) naphtho-γ-pyrones extracted from the molecular networking (MN) of *P. influorescens* and *P. nobilis* co-culture (Figure 1F). Red nodes represent peak ions from the extract of *P. influorescens* mono-culture and blue nodes represent peak ions produced only in the co-culture.

As displayed in the UPLC chromatogram (Figure 2), two distinct major peaks (compounds **1**/**2**, *t*_R_ 3.38 and **5**, *t*_R_ 7.37) were observed in the whole co-culture. Hence, these peaks were prioritized for isolation and chemical characterization. Large-scale cultivation of the co-culture of *P. influorescens* and *P. nobilis* on 200 PDA plates was carried out. The crude EtOAc extract was sequentially partitioned by a modified Kupchan extraction approach [25] to afford *n*-hexane, dichloromethane (DCM) and aqueous MeOH subextracts. The DCM subextract that exhibited the highest activity against the phytopathogen *X. campestris* (Table S2) contained the prioritized compounds (**1**, **2** and **5**). An in-depth analysis of the MN of the crude extract of *P. influorescens* with *P. nobilis* (Figure 3), allowed to track these peaks down to two molecular clusters, i.e. cyclic macrolactones (compounds **1** and **2**, Figure 3 and Figure 4A) and naphtho-γ-pyrones (compound **5**, Figure 3 and Figure 4B) respectively. The confrontation zone (Figure 2B, iv) contained high levels of compound **4**. The inspection of the MN indicated that **4** belonged to a small azaphilone cluster, where two nodes representing compounds **3** and **4** were the main constituents. Therefore, we decided to isolate these compounds as well.

As shown in Figure 4A, the cyclic macrolactone cluster (A) revealed four nodes (**a**–**d**) representing four peak ions. The first node (a) *m/z* 251.0831 [M + Na]^+^ was dereplicated as the polyketide dendrodolide L [26] by searching the predicted molecular formula C_12_H_20_O_4_Na against multiple online and commercial databases. Predicted molecular formulae for all other nodes in this cluster returned no hits with a macrolactone scaffold, hence are putatively new. The node (b) *m/z* 249.1106 [M + Na]^+^ represented the new compound **2**. Node (c) *m/z* 253.0831 [M + Na]^+^ had a mass difference of 2 amu from node (a), which is indicative of an unsaturation. We tentatively proposed a structure for (c) (Figure 4A, c) based on the high spectral similarity score (>0.7) to (b) as implemented in the GNPS-MN platform (see MN in experimental section). Nodes (c) and (d) (*m/z* 367.1570 [M + Na]^+^) were not purified because they were present only in very minute quantities. The other two nodes 254.094 and 252.079 are isotopic nodes for (c) and (a) respectively. In addition, we purified dendrodolide E (**1**) [26], which was not detected in the MN due to the low intensity of its fragment ions.

Naphtho-γ-pyrone cluster (B) comprised of compounds that were only expressed in the co-culture. Within the cluster B (Figure 4B), the node (a), *m/z* 519.1273 [M + H]^+^ was the main *bis*-naphtho-γ-pyrone cephalochromin (**5**) produced in the co-culture. HPLC-ESI-MS extracted ion current analysis showed that compound **5** was a minor component of the *P. influorescens* mono-culture extract. The biosynthesis of compound **5** in the co-culture was enhanced approximately 12-fold, based on the observed peak ion intensities of **5** in the co-culture and the mono-culture. Node (b), *m/z* 517.0802 [M + H]^+^ was annotated as ustilaginoidin G (the dehydro derivative of cephalochromin). The last node (c), *m/z* 521.1382 [M + H]^+^ with a predicted molecular formula of C_28_H_25_O_10_, returned no hit and may represent a putatively new naphtho-γ-pyrone derivative. However, it could not be purified due to its insufficient quantity. The other two nodes with *m/z* 518.087 and *m/z* 520.101 are isotopic nodes for (b) and (c), respectively.

As mentioned above, another MF observed in the MN belonged to azaphilone class. A detailed MN analysis (Figure 3) revealed a close relationship of two nodes within this cluster (compounds **3** and **4** with their isotopic nodes) that were also produced in the co-culture. Hence, we purified compounds **3** and **4**, since the UPLC chromatogram (Figure 2B) showed the specific presence of compound **3** in the whole co-culture (minor compound) and compound **4** in the confrontation zone (one of the major compounds). They were identified as the known polyketide spiciferinone (**3**) and its new derivative 8a-hydroxy-spiciferinone (**4**).

**Figure 5:**
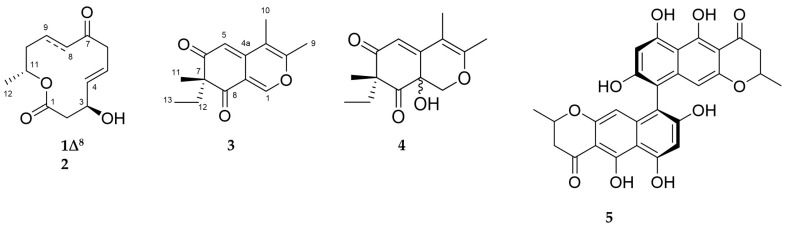
Chemical structures of compounds **1**–**5**.

Compound **1** was isolated as a colorless film with the molecular formula C_12_H_16_O_4_ deduced by HRESIMS. Its ^1^H and ^13^C NMR data (Figures S7–S10) were identical to those of dendrodolide E, a 12-membered macrolide previously reported from the fungus *Dendrodochium* sp. associated with the sea cucumber *Holothuria nobilis* [26]. Compound **1** exhibited the same sign of specific rotation value [α]20D +80, *c* 0.005, CHCl_3_) as (+)−dendrodolide E ([α]${}_{D}^{27}$ +42.7, *c* 0.09, CHCl_3_) [26], hence identified as (+)*-*(3*S*,11*R*)-dendrodolide E (Figure 5).

**Figure 6:**
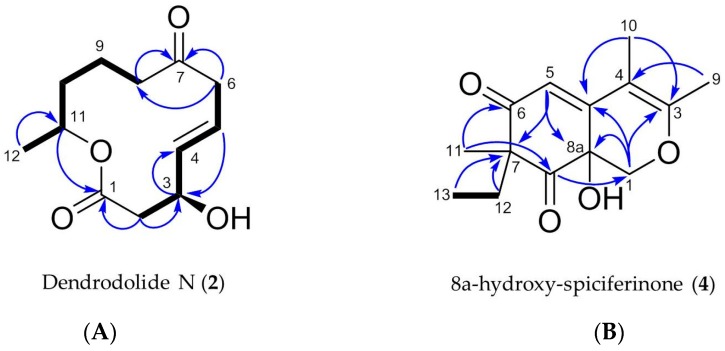
Key COSY (in bold) and HMBC correlations (blue arrows) observed for (**A**) compound **2** and (**B**) compound **4**.

Dendrodolide N (**2**) was obtained as an optically active colorless solid. Based on its HRESIMS data (*m/z* 249.1106 [M + Na]^+^), its molecular formula was identified as C_12_H_18_O_4_, indicating four degrees of unsaturation (DoU). The FT-IR spectrum displayed intense absorption bands at ν_max_ 3409 (O-H stretch), 1707 (ester C=O stretch) and 1671 (ketone C=O stretch) cm^−1^. The ^13^C NMR spectrum contained twelve carbon resonances, which were categorized into two carbonyl groups (*δ*_C_ 171.9, and 212.4), two sp^2^-hybridized methines (*δ*_C_ 137.7, 124.7), five methylenes (*δ*_C_ 20.8, 34.5, 42.8, 43.1, 47.4), two oxymethines (*δ*_C_ 69.0, 71.1), and one methyl (*δ*_C_ 20.1) carbons. This data corresponded to three DoU, indicating **2** to be a monocyclic compound. The ^1^HNMR spectrum included resonances characteristic for two olefinic methines (*δ*_H_ 5.83, dd, *J* = 15.4, 4.8 Hz and *δ*_H_ 5.73, m), two oxymethines (*δ*_H_ 4.53, m and *δ*_H_ 4.97, m), a secondary methyl (*δ*_H_ 1.22, d, *J* = 6.3 Hz), and five methylene protons (*δ*_H_ between 1.54–3.22) (Table 2, Figures S11–S19). The 2D COSY spectrum of **2** revealed the presence of two clear spin systems (Figure 6A). One spin system consisted of the secondary methyl group H_3_-12 (*δ*_H_ 1.22), the oxymethine proton H-11 (*δ*_H_ 4.97), and three methylene protons: H_2_-10 (*δ*_H_ 1.54), H_2_-9 (*δ*_H_ 1.94 and *δ*_H_ 1.64), and H_2_-8 (*δ*_H_ 2.3 and *δ*_H_ 2.5). The other spin network started from H_2_-2 (*δ*_H_ 2.72 and *δ*_H_ 2.57) that coupled with the oxymethine proton H-3 (*δ*_H_ 4.53). The latter proton had a scalar coupling with the olefinic proton H-4 (*δ*_H_ 5.83) which in turn coupled with the second olefinic methine H-5, (*δ*_H_ 5.73). Finally, H-5 was coupled with the methylene protons of H_2_-6 (*δ*_H_ 3.22 and *δ*_H_ 3.05) to finalize the spin network. The two spin systems were readily connected to each other with the aid of ^1^H-^13^C long-range correlations observed in the HMBC spectrum (Figure 6A). The HMBC correlations between H_2_-8/C-7, H_2_-6/C-7, and H_2_-6/C-8 completed the upper end of the molecule (from C-8 to C-6). Diagnostic HMBC correlations from both H_2_-2 and H-11 to the ester carbonyl C-1 (*δ*_C_ 171.9) linked C-11 and C-2 thorough C-1, thereby establishing the full macrocyclic ring. The hydroxyl group was assigned to C-3 (*δ*_C_ 69.0), due to additional HMBC correlations observed between H-2/C-3, H-3/C-4, H-4/C-3 and H-5/C-3. The geometry of the double bond at C-4 was determined as *E*, due to the large coupling constant value (*J*_4,5_ = 15.4 Hz). Thus, **2** was assigned the planar structure as shown in Figure 6A, which is the C-8/C-9 saturated derivative of compound **1**.

The relative configuration of the stereogenic centers C-3 and C-11 was assigned on the basis of an NOESY experiment. A NOESY correlation between H-3 and H-5 suggested the proximity of both protons, and suggesting an α-orientation for H-3 [26]. Further NOESY correlations observed between H-8*β*/6*β* and H-8*β*/H-11 suggested H-11 to be *β* oriented. These data indicated that 2 is a new analogue of the dendrodolide class of compounds [26]. Based on the NOE data and the measured specific rotation value (+146, *c* 0.0085, CHCl_3_), we suggest the trivial name (+)-(3*S*,11*R*)- dendrodolide N for compound **2**.

Compound **3** was isolated as a yellowish amorphous powder with a molecular formula of C_14_H_16_O_3_ assigned by HRESIMS. Further analysis of its 1D NMR and 2D NMR data (Table 3, Figures S20–S25), led to identification of **3** as spiciferinone, an azaphilone type phytotoxin that was reported from the terrestrial fungus *Cochliobolus spicifer* [27]. The measured specific rotation value of **3** [α]${}_{D}^{20}$ +36, *c* 0.001, MeOH) was similar to that reported in the literature ([α]${}_{D}^{20}$ +63, *c* 0.1, MeOH) [27].

The molecular formula C_14_H_18_O_4_ (*m/z* 251.1285 [M + H]^+^) was assigned to compound **4** by HRESIMS, indicating the presence of six DoU. The ^1^H NMR spectrum of **4** possessed resonances for an isolated olefinic proton (H-5, *δ*_H_ 5.87 s), one oxymethylene (H-1, *δ*_H_ 4.48 d, and *δ*_H_ 3.84 d), two olefinic methyl groups (H_3_-9, *δ*_H_ 2.08 s, H_3_-10 *δ*_H_ 1.87 s), one tertiary methyl (H_3_-11, *δ*_H_ 1.19 s), plus an ethyl function (H_2_-12, *δ*_H_ 2.27 dq and *δ*_H_ 1.80 dq; H_3_-13, *δ*_H_ 0.88 t) (Table 3, Figures S26–34). From the ^13^C NMR spectrum, it was evident that **4** contained two carbonyl functions (C-6 *δ*_C_ 201.9 and C-8 *δ*_C_ 207.4), two double bonds (C-3 *δ*_C_ 161.8, C-4 *δ*_C_ 104.3, C-4a *δ*_C_ 152.3 and C-5 *δ*_C_ 116.0), and two oxygenated carbons (C-1 *δ*_C_ 70.8, C-8a *δ*_C_ 67.2) (Table 3). This data was indicative of a bicyclic azaphilone structure, as found in compound **3**. Comparison of the 1D and 2D NMR data of **4** suggested a close resemblance to compound **3.** First major difference between **3** and **4** was the absence of the double bond at ∆^1(8a)^, hence the olefinic proton H-1 was replaced with an oxymethylene group (*δ*_H_ 4.48, d, *J* = 12.4 Hz and 3.84, d, *J* = 12.4 Hz) in **4** (Table 3). Secondly, the molecular formula of **4** was 18 atomic mass units higher than **3**, suggesting the presence of a hydroxyl substituent in **4**. This assumption was further supported by a broad absorption band observed in the FT-IR spectrum of **4** at ν_max_ 3220 cm^−1^. The hydroxyl group was assigned to C-8a, based on its ^13^C chemical shift (*δ*_C_ 67.2), plus HMBC correlations detected between H-1/C-8a and H-5/C-8a, finalizing the planar structure of **4** (Figure 6B). The specific rotation of **4** ([α]${}_{D}^{20}$ +75, *c* 0.0006, MeOH) was similar to that of **3**. Given the similarity in specific rotation, structure and same biological source, it is biosynthetically reasonable to assume that **4** has the same *(R*) configuration at C-7. However, we were unable to assign the configuration of C-8a. We suggest the trivial name of 8a-hydroxy-spiciferinone for compound **4**.

Compound **5** was isolated as an optically active yellow powder [α]${}_{D}^{20}$ +468, *c* 0.0008, CHCl_3_) with a molecular formula C_28_H_23_O_9_ deduced by HRESIMS. Analysis of the ^1^H NMR, ^13^C NMR, HRMS data, and [α]${}_{D}^{20}$ value (Figures S35–S38) suggested **5** to be cephalochromin, a *bis*-naphtho-γ-pyrone that was previously isolated from many fungi, including *Pseudoanguillospora* sp. and *Ustilaginoidea virens* [28,29].

### 2.4. Anti-Phytopathogenic Activity of Isolated Compounds

All isolated compounds were tested in vitro against a panel of six phytopathogens, except for compound 1, which was obtained in very low amounts. Compound 5 that was specifically overexpressed in the co-culture exhibited the highest activity against *X. campestris* and *P. infestans* with IC_50_ values of 0.9 µg/mL and 1.7 µg/mL, respectively (Table 4). The new compound dendrodolide N (2) showed moderate activity against *P. infestans* (IC_50_ 13.9 µg/mL, Table 4), but was inactive against all other pathogens at the highest test concentration (100 µg/mL). Compounds 3 and 4 showed no activity against any of the plant pathogens within the range of concentrations tested.

## 3. Discussion

Co-cultivation is a cheap and straightforward approach, requiring regulatory mechanisms involving genetic and molecular information transfer to produce secondary metabolites [30]. Production of these metabolites is complex, dynamic, and unforeseeable, although it is the driving force for microbial survival in complex multispecies habitats. In microbial drug discovery, co-cultivation of microorganisms from the same environment represents an ecology-inspired approach to activate cryptic gene clusters resulting in biosynthesis of novel molecules, new analogs of known compounds and/or overexpression of previously known molecules [31]. However, co-cultivation may also lead to competitive behavior, thereby causing suppression of gene clusters in competing strains [32]. A recent study revealed that microbes respond to cues produced by competitors by suppressing antibiotic synthesis in competitors in order to reduce threats to themselves [33]. Co-cultivation experiments often follow different approaches including painstaking bipartite screening of a collection of microbes of the same or different genera, niches or environment [34]. Researchers also tend to co-cultivate well-studied species with other microbes, introducing a level of bias [19]. However, these approaches are rather time-consuming and untargeted for selection of the best co-cultivation pairs.

In this study, intra- and inter-categorial (strong and weak) solid-state co-cultivation allowed a direct visualization of the interactions on the plates. With the exception of *P. influorescens* (strong)–*Lentithecium sp.* (weak) co-culture, which showed slight mycelial contact (Figure 1E), all co-cultures of strong candidates (Helotiales sp. and *P. influorescens*) displayed patterns of distance inhibition. This pattern is associated with the release of antimicrobial molecules into the medium to inhibit competitors [35,36]. In contrast, the co-culture of the weak candidates (*P. nobilis* and *Lentithecium* sp.) displayed mutual interaction [37] as fungal mycelia grew freely into each other.

A comparative metabolomics approach used in this study revealed the production of minor new metabolites (observed as blue nodes in MNs, Figures S2–S6) in all co-cultures, irrespective of the visual interaction type. This is in line with findings of previous studies reporting a non-significant difference in the number of induced metabolites in the confrontation areas of 600 co-cultures exhibiting different interaction types [36]. However, due to their low quantity, the induced metabolites were not detected in significant amounts in the UPLC chromatograms of the co-cultures. The co-culture of *P. influorescens* (strong)—*P. nobilis* (weak) was selected for large-scale cultivation and chemical analysis because of: (a) the significantly different metabolite profile compared to the axenic cultures; (b) the observation of a co-culture specific cluster in the MN (identified as the *bis*-naphtho-γ-pyrone cluster); and (c) the enhanced activity of co-culture against the phytopathogen *X. campestris* (Table 1). The chemical profile of *P. influorescens* (strong)—*P. nobilis* (weak) co-culture showed a marked reduction in the chemical diversity compared to their respective mono-cultures. The biosynthesis of numerous compound clusters observed in the single fungal cultures (dereplicated as terpenes and isocoumarins) were suppressed, while the *bis*-naphtho-γ-pyrone type mycotoxin cephalochromin (**5**) was upregulated in *P. influorescens*. An earlier report involving the co-cultivation of mycoparasite *Stachybotrys elegans* and the soil-borne fungal pathogen *Rhizoctonia solani* revealed that the mycotoxins produced by the mycoparasite altered the metabolism of the fungal pathogen. The biosynthesis of many antimicrobial compounds in *R. solani* was downregulated, while trichothecene type mycotoxins were upregulated in *S. elegans* [32]. In this study, chemical signals produced by *P. nobilis* appeared to have activated PKS genes (non-reducing polyketide synthase [38]) responsible for cephalochromin biosynthesis in *P. influorescens*. Cephalochromin has previously been isolated from several fungi including *Cosmospora vilior*, *Cephalosporium* sp., *Pseudoanguillospora* sp., and *Verticillium* sp. [39]. It is however being reported/detected in the genus *Plenodomus* for the first time. Consistent with our bioactivity data (IC_50_ value 1.7 µg/mL), cephalochromin has been shown to exert anti-phytopathogenic activity against the oomycete *P. infestans* [28]. Its activity against the phytopathogenic bacterium *X. campestris* (IC_50_ value 0.9 µg/mL) is being reported for the first time herein.

Dendrodolides E (**1**) and N (**2**) were observed in the monoculture of *P. influorescens* and also produced in the co-culture. They belong to the 12-membered macrolide family, commonly produced in fungi with antimicrobial and cytotoxic activities [40,41,42]. Their biosynthesis involves a highly reducing polyketide synthase from a hexaketide starter unit with a cyclization of their linear chain elongation [26,43]. The dendrodolides have been reported as a subfamily of the 12-membered ring macrolides from the fungus *Dendrodochium* sp. isolated from a sea cucumber *Holothuria nobilis* Selanka [26]. The current study adds another new member to this subfamily as dendrodolide N, with moderate inhibitory activity (IC_50_ value 13.9 µg/mL) against the phytopathogenic oomycete *P. infestans*. It is the first report of such macrolides from the fungal genus *Plenodomus.*

Spiciferinone (**3**) and its new hydroxylated derivative (**4**) belong to the rare class of azaphilone type polyketides with a meta-quinone ring. They were produced in the monoculture of *P. nobilis* and detected in comparatively smaller amounts in the co-culture. Spiciferinone (**3**) was previously characterized as a phytotoxin produced by a terrestrial fungus *Cochliobolus spicifer* [27]. It is the first observation of such azaphilones in the fungal genus *Pyrenochaeta.* In our bioactivity tests against bacterial and fungal phytopathogens, spiciferinone (**3**), as well as its new derivative (**4**), remained inactive. Azaphilones display a wide range of activities including antiviral, anti-inflammatory, anticancer and antioxidant among others [44], thereby warranting further bioactivity investigations.

In summary, we employed a new, alternative co-cultivation approach, which exploits the anti-phytopathogenic potency of axenic cultures as proxies to categorize and subsequently select co-culture partners by comparative MN and bioactivity assessments. Our results describe an example of biosynthetic gene suppression in one strain and significant induction of a known mycotoxin in another strain simultaneously. Our future efforts will include identifying the chemical signals responsible for overexpression of polyketides, such as cephalochromin and characterizing the individual metabolites of the mono-cultures. Genome analysis will also be included to understand the influence of co-cultivation on the expression of functional metabolites in these two fungi.

## 4. Materials and Methods

### 4.1. General Procedures

Specific rotations of compounds **1**–**5** were measured on a monochromatic light source in MeOH or CHCl_3_ at 20 °C on a Jasco P-2000 polarimeter (Jasco, Pfungstadt, Germany). IR spectra were recorded on a PerkinElmer Spectrum Two FT-IR spectrometer (PerkinElmer, Boston, MA, USA). HRMS was recorded on micrOTOF II-High-performance TOF-MS system (Bruker^®^, Billerica, MA, USA) equipped with an electrospray ionization source. The NMR spectra were acquired on a BRUKER AV 600 spectrometer equipped with a Z-gradient triple resonance cryo-probehead (Bruker^®^, Billerica, MA, USA). All spectra were run in solvents as specified in the text with referencing to residual ^1^H and ^13^C signals in the deuterated solvent. HPLC separations were performed on an Acquity UPLC HSS T3 column (High Strength Silica C18, 1.8 mm, 100 2.1 mm, Waters, Milford, MA, USA). HRMS/MS data were recorded on an Acquity UPLC I-Class System coupled to a Xevo G2-XS QToF Mass Spectrometer (Waters^®^, Milford, MA, USA) in the positive mode at a mass range of *m/z* 50–1600 Da. Semi-preparative HPLC separations were performed using a VWR Hitachi Chromaster system (VWR International, Allison Park, PA, USA) consisting of a 5310 column oven, a 5260 autosampler, a 5110 pump, and a 5430 diode array detector connected in parallel with a VWR Evaporative Light Scattering Detector (ELSD 90, VWR International, Allison Park, PA, USA). Separation was achieved using a C18 semi-preparative column (Onyx, 10 mm × 100 mm, Phenomenex, Torrance, CA, USA) or on a Synergi 4u Polar-RP 80A column (250 × 4.6 mm).

### 4.2. Fungal Materials

All eight fungi were isolated from sediment samples obtained from the Windebyer Noor, Schleswig-Holstein, Germany in November 2015 [21]. Identification of the fungal strains by Sanger sequencing of the internal transcribed spacer (ITS) regions or 18S rRNA gene was reported previously [21]. Isolates used in this study had GenBank accession numbers MH791233 (ITS sequence), MH791253 (ITS sequence), MH791254 (ITS sequence), MH791292 (ITS sequence), MH791174 (18S rRNA gene sequence), MH791258 (ITS sequence), MH791244 (ITS sequence), and MH791275 (ITS sequence). The fungal strains were identified at species, genus or order level based on their closest relative according to BLAST [45]. To allow an assignment to the highest possible taxonomic rank, two new BLAST searches (access of NCBI nucleotide database: 31.07.19, Table S1) were conducted for all eight strains, one searching for highly similar sequences and the second searching for sequences from type material only. The combined information from both searches allowed four cases of assignment to the species level, to the genus level in one case, and to order level only in three cases. Fungi were cryopreserved in liquid nitrogen using Microbank TM (PRO-LAB Diagnostics, Richmond Hill, Canada). Each fungal culture was maintained on potato dextrose agar (PDA-potato starch 4 g, glucose monohydrate 4 g, agar 15 g, and dH2O ad 1 L) at 22 °C.

### 4.3. Fungal Cultures and Extraction

Fungal strains were grown on PDA medium at 22 °C in the dark for two weeks as pre-cultures. Axenic cultures were prepared by inoculating 5 mm agar plug from pre-cultures on PDA medium in 9 cm petri dishes. Each strain was cultivated in triplicate at 22 °C for 21 days. Each replicate was grown on five Petri dishes to ensure sufficient yield of extracts for analyses. Similarly, co-culture experiments were conducted by inoculating 5 mm plugs of pre-cultures of competing strains on opposite sides of a petri dish (about 3 cm apart). Co-cultures were incubated at the same conditions as the mono-cultures.

Initially, a small-scale extraction of the mono- and co-cultures was performed. Whole cultures (5 PDA plates per culture) were sliced into pieces and extracted with EtOAc (2 × 200 mL) (PESTINORM, VWR Chemicals, Leuven, Belgium) after homogenizing by an Ultra-Turrax at 19000 rpm. The extract was filtered through a cellulose membrane filter (type 113P, Rotilabo^®^ ROTH, Karlsruhe). The filtrate was collected in a separating funnel and washed twice with 100 mL of Milli-Q^®^ water (Arium^®^ Lab water systems, Sartorius) to remove salts and water-soluble compounds. The organic phase was collected and evaporated under reduced pressure (200 bar, 150 rpm at 40 °C). Dried extracts were re-dissolved in 3 mL of ULC/MS grade methanol (MeOH) and pipetted into a pre-weighed 4 mL amber glass vial through a 13 mm syringe filter with 0.2 µm PTFE membrane (VWR International, Darmstadt, Germany). The extracts were dried under a nitrogen blow and stored at −20 °C.

### 4.4. UPLC-QToF-MS Analysis

Chromatograms were acquired on the UPLC I-Class System coupled to a Xevo G2-XS QToF Mass Spectrometer (Waters^®^, Milford, MA, USA) at a concentration of 0.1 mg/mL. Sample separation was achieved on an Acquity UPLC HSS T3 column (High Strength Silica C18, 1.8 µm, 100 × 2.1 mm, Waters^®^) at 40 °C at a flow rate of 0.6 mL/min and injection volume of 2 µL with a binary solvent system comprised of a mobile phase A: 99.9% MilliQ^®^-water/0.1% formic acid (ULC/MS grade) and phase B: 99.9% acetonitrile (MeCN, ULC/MS grade, Biosolve BV, Dieuze, France) / 0.1% formic acid in a linear gradient: 99% A for 11.5 min, 0% A for 1 min and 99% A until minute 15.

MS and MS/MS fragmentation spectra were acquired in a data dependent analysis (DDA) mode with an electrospray ionization source in the positive mode using the following parameters: A mass range of *m/z* 50–1600 Da, capillary voltage of 0.8 KV, cone and desolvation gas flow of 50 and 1200 L/h, respectively, source temperature at 150 °C and desolvation temperature at 550 °C with sampling cone and source offset at 40 and 80, respectively. Collision energy (CE) was ramped: Low CE from 6–60 eV to high CE of 9–80 eV. As controls, solvent (MeOH) and non-inoculated medium were injected under the same conditions.

### 4.5. Molecular Networking

Data obtained from the UPLC-MS/MS system were converted a to mzXML format using Proteo Wizard msconvert (version 3.0.10051, Vanderbilt University, Nashville, TN, USA) [46], uploaded to Global Natural Products Social Molecular Networking (GNPS) platform, and analyzed using the molecular networking workflow (http://gnps.ucsd.edu). The data were filtered by removing all MS/MS peaks within +/− 17 Da of the precursor *m/z*. MS/MS spectra were window filtered by choosing only the top six peaks in the +/− 50 Da window throughout the spectrum. The data were clustered with MS-Cluster with a parent mass tolerance and MS/MS fragment ion tolerance 0.02 Da. Further, consensus spectra that contained less than two spectra were discarded. A network was then created where edges were filtered to have a cosine score above 0.7 and more than six matched peaks. Further edges between two nodes were kept in the network only if each of the nodes appeared in each other’s respective top 10 most similar nodes. The spectra in the network were then searched against GNPS spectral libraries. The library spectra were filtered in the same manner as the input data. All matches kept between network spectra and library spectra were required to have a score above 0.7 and at least 6 matched peaks [47]. The MS/MS spectra were then searched against an in-silico MS/MS database (ISDB) using the fragmentation tool CFM-ID [48]. The molecular networking data were visualized in Cytoscape 3.5.1. program using ‘directed ‘style [49]. All compounds (nodes) originating from PDA media and solvent control (MeOH) were deleted from the original network enabling visualization of metabolites coming from mono- and co-cultures.

Manual annotation of peak ions was performed using MassLynx^®^ version 4.1. by searching their predicted molecular formula against databases such us MarinLit, Reaxys, Dictionary of Natural Products and PubChem. All hits were validated based on their biological source and fragmentation patterns using the CFM-ID web server [48]. The molecular networking jobs on GNPS can be found at https://gnps.ucsd.edu/ProteoSAFe/status.jsp?task=96db257f75f24bf799f861327b04a995 (co-culture Helotiales sp. and *P. influorescens*), https://gnps.ucsd.edu/ProteoSAFe/status.jsp?task=30eb31eeca824e1cb546a0bd860865dc (co-culture *P. nobilis* and *Lentithecium* sp.) https://gnps.ucsd.edu/ProteoSAFe/status.jsp?task=950576598be541eca730f5f774597d12 (*P. influorescens* and *Lentithecium* sp.); https://gnps.ucsd.edu/ProteoSAFe/status.jsp?task=3c80796e247b4712b97877d08e38c11b (co-culture Helotiales sp. and *P. nobilis*); https://gnps.ucsd.edu/ProteoSAFe/status.jsp?task=70126f393fd0498db4d1af1c11948c30 (co-culture *P. influorescens* and *P. nobilis*); https://gnps.ucsd.edu/ProteoSAFe/status.jsp?task=02dd864c69a442f6ad785cb54c941d67 (co-culture Helotiales sp. and *Lentithecium* sp.) The annotated MS/MS spectra were deposited in the GNPS spectral library under references CCMSLIB00005490881, CCMSLIB00005490882 and CCMSLIB00005490883 for compounds **2**–**4**, respectively.

### 4.6. Isolation of Compounds ***1***–***5***

Pre-cultures of *P. influorescens* and *P. nobilis* were grown for 14 days at 22 °C on a PDA medium. Mycelial plugs (1 cm^2^) of both pre-cultures were transferred onto a PDA plate at 5 cm apart. For large scale cultivation, 200 PDA plates were inoculated and incubated at 22 °C for 21 days. The same extraction protocol described in Section 4.3 was used to yield a pooled EtOAc extract (1.2 g). The crude extract was partitioned using the modified Kupchan solvent-solvent extraction [25] to yield *n*-hexane (333 mg), DCM (520 mg) and aq. MeOH (9 mg) subextracts. Briefly, the crude extract was first partitioned between 10% aqueous MeOH (100 mL) and *n*-hexane in a ratio of 1:1 (3 × 100 mL). The water concentration of the aq. MeOH phase was increased to 35%, before partitioning against DCM in a ratio of 1:1 (3 × 140 mL). The best anti-phytopathogenic activity was observed with the DCM subextract (Table S2). The DCM subextract was fractionated on a Chromabond SPE C18 column cartridge (Macherey-Nagel, Duren, Germany) using a step gradient MeOH in water (0% to 100%) to yield 11 fractions. The highest anti-phytopathogenic activity was tracked to fractions 7–9 (Table S2). They were combined (189 mg) and subjected to semi-prep. RP-HPLC with a gradient of H_2_O:MeCN from 42:58 to 0:100 in 15 min to yield an impure fraction. This fraction was rechromatographed on an analytical column by RP-HPLC with an isocratic mixture of H_2_O:MeCN (40:60 in 15 min, flow 1 mL/min) to yield **5** (10 mg, t_R_ 9 min). The combined SPE fractions 3–6 (120 mg) with moderate activities were repeatedly purified by RP-HPLC on an analytical column to furnish **1** (0.7 mg) and **2** (2.6 mg), along with **3** (1.5 mg) and **4** (2.0 mg).

*(+)-(3S, 11R)-Dendrodolide E* (**1**): Colorless film; ${\left[\alpha \right]}_{D}^{20}$ +80 (*c* 0.005, CHCl_3_). ^1^H NMR (CDCl_3_, 600 MHz) and ^13^C NMR data (CDCl_3_, 150 MHz), see Figures S7–S9; (+)*-*HRESIMS *m/z* 247.0937 [M + Na]+ (calculated for C_12_H_16_O_4_Na, *m/z* 247.0941).

*(+)-(3R, 11R)-Dendrodolide N* (**2**): Colorless film; ${\left[\alpha \right]}_{D}^{20}$ +146 (*c* 0.0085, CHCl_3_). IR (film) ν_max_, 3409 (br), 2919, 1707, 1671, 1598, 1453, 1362, 1271, 1176 cm^−1^. ^1^H NMR (CD_3_OD, 600 MHz) and ^13^C NMR data (CD_3_OD, 150 MHz), Table 2; (+)-HRESIMS *m/z* 249.1106 [M + Na]+ (calculated for C_12_H_18_O_4_Na, *m/z* 249.1103).

*(+)-(R)-Spiciferinone* (**3**): Yellowish amorphous solid; ${\left[\alpha \right]}_{D}^{20}$ +36 (*c* 0.001, MeOH). For ^1^H NMR (CDCl_3_, 600 MHz) and ^13^C NMR data (CDCl_3_, 150 MHz), Table 3; (+)*-*HRESIMS *m/z* 233.1177 [M + H]^+^ (calculated for C_14_H_17_O_3_, *m/z* 233.1178).

*(+)-(7R)-8a-Hydroxy-spiciferinone* (**4**): Yellowish amorphous solid; ${\left[\alpha \right]}_{D}^{20}$ +75 (*c* 0.0006, MeOH). IR (film) ν_max_, 3220 (br), 2924, 2854, 1715, 1630, 1577, 1207, 1152 cm^−1^. ^1^H NMR (CD_3_OD, 600 MHz) and ^13^C NMR data (CD_3_OD, 150 MHz), Table 3; (+)*-*HRESIMS *m/z* 251.1285 [M + H]^+^ (calculated for C_14_H_19_O_4_, *m/z* 251.1283).

*Cephalochromin* (**5**): Yellowish amorphous powder; ${\left[\alpha \right]}_{D}^{20}$ +468 (*c* 0.0008, CHCl_3_). For ^1^H NMR (CDCl_3_, 600 MHz) and ^13^C NMR data (CDCl_3_, 150 MHz), see Figures S35–S37; (+)*-*HRESIMS *m/z* 519.1293 [M + H]^+^ (calculated for C_28_H_23_O_10_, *m/z* 519.1291).

### 4.7. Bioactivity

Plant pathogens used as test strains for bioactivity assays were *Erwinia amylovora* DSM 50901, *Pseudomonas syringae* pv. aptata DSM 50252, *Ralstonia solanacearum* DSM 9544, *Xanthomonas campestris* DSM 2405, and *Magnaporthe oryzae* DSM 62938 purchased from Deutsche Sammlung für Mikroorganismen und Zellkulturen (DSMZ, Braunschweig, Germany), whereas *Phytophthora infestans* CBS 120920 was purchased from Westerdijk Fungal Biodiversity Institute/Centraal bureau voor Schimmelcultures (Utrecht, Netherlands). Extracts were tested in triplicates against the phytopathogens. Bioactivity testing procedure has been described before [21]. Briefly, the broth dilution approach in 96-well microplate was employed. All crude extracts were dissolved in DMSO and added to the test organisms in 96 well microplates to make an effective test concentration of 100 μg/mL and 0.5% (*v/v*) DMSO. Several antibiotics (chloramphenicol, tetracycline, nystatin, and cycloheximide) served as positive controls, whereas the growth medium (PDA) and 0.5% (*v/v*) DMSO were tested as negative controls. Absorbances were measured at 600 nm using an Infinite M200 reader (TECAN Deutschland GmbH, Crailsheim, Germany) before and after incubation and % inhibition values computed [21]. The IC_50_ values of the pure compounds were calculated using the Microsoft Excel program.

## Figures and Tables

**Table 1:** Anti-phytopathogenic activity (%) of the fungal mono- and co-culture extracts at a test concentration of 100 µg/mL against Ps: *P. syringae*; Xc: *X. campestris*; Ea: *E. amylovora*; Rs: *R. solanacearum*; Mo: *M. oryzae*; Pi: *P. infestans*). The letters ‘w’ and ‘s’ represent weak and strong activity, respectively. Strong activities are shown in bold letters. Reference compound for Xc, Ea, and Ps: 10 µM chloramphenicol; Rs: 10 µM tetracycline, Mo: 10 µM nystatin, Pi: 10 µM cycloheximide.

Strain	Category	Ps	Xc	Ea	Rs	Mo	Pi
**Mono-cultures**							
Helotiales sp.	s	31	**83**	55	27	**99**	**92**
*Plenodomus influorescens*	s	-	-	-	-	**99**	**93**
*Penicillium bialowiezense* ^a^	s	-	-	-	-	**89**	**91**
*Sarocladium strictum* ^a^	w	-	-	-	-	-	57
*Pyrenochaeta* sp.*^,a^	w	-	-	-	-	-	75
*Pyrenochaeta nobilis*	w	-	-	-	-	38	**89**
*Lentithecium* sp.	w	-	-	-	-	-	25
*Lentithecium* sp. ^a^	w	-	-	-	-	-	35
**Co-cultures**							
Helotiales_*P. influorescens*	s/s	-	99	22	-	95	79
Helotiales_ *P. nobilis*	s/w	-	21	-	34	94	66
Helotiales_*Lentithecium*	s/w	-	40	-	-	96	75
*P. influorescens*_*P. nobilis*	s/w	-	59	-	-	90	97
*P. influorescens*_*P. nobilis*^b^	s/w	-	90	-	-	34	81
*P. influorescens*_*Lentithecium*	s/w	-	21	-	-	91	95
*P. nobilis*_*Lentithecium*	w/w	-	35	-	-	72	98
Reference compound		98	95	97	89	100	92

* A new BLAST search (13-08-2019) revealed a related strain *Neocucurbitaria* sp. (Table S1). ^a^ Strains excluded from co-cultivation study; ^b^ confrontation zone.

**Table 2:** ^1^H NMR data of compounds **1** and **2** (600 MHz, acquired in ^a^ CDCl_3_ or ^b^ CD_3_OD).

Position	1 ^a^	2 ^a^	2 ^b^	
	*δ*_H_, Mult. (*J* in Hz)	*δ*_H_, Mult. (*J* in Hz)	*δ*_H_, Mult. (*J* in Hz)	*δ*_C_ (Type)
1				171.9, C
2 α	2.77 dd (14.0, 4.8)	2.73 dd (13.3, 5.2)	2.72 dd (13.4, 3.9)	43.1, CH_2_
β	2.51 dd (14.0,2.9)	2.61 dd (13.3, 3.2)	2.57 dd (13.4, 6.6)	
3	4.52 s	4.58 s	4.53 m	69.0, CH
4	5.58 dd (15.8, 1.5)	5.80 m	5.83 dd (15.4, 4.8)	137.7, CH
5	5.77 m	5.80 m	5.73 m	124.7, CH
6 α	3.22 dd (14.1, 5.6)	3.18 dd (14.7, 8.9)	3.22 dd (13.5, 8.3)	47.4, CH_2_
β	3.32dd (14.1, 8.0)	3.03 m	3.05 dd (13.6, 66)	
7				212.4, C
8 α	5.96 d (16.2)	2.21 m	2.3 dt (12.9, 7.6)	42.8, CH_2_
β		2.46 m	2.5 m	
9 α	6.54 ddd (16.2, 9.0, 7.0)	1.84 m	1.94 ddt (15, 13.4, 6.6)	20.8, CH_2_
β		1.59 m	1.64 m	
10 α	2.29 dddd (14.0, 11.2, 8.9, 0.9)	1.59 m	1.54 m	34.5, CH_2_
β	2.42 dddd (14.0, 6.3, 3, 1.4)			
11	5.27 m	5.1 m	4.97 m	71.1, CH
12	1.32, d (6.3)	1.23 d (6.4)	1.22 d (6.3)	20.1, CH_3_

**Table 3:** NMR data of compounds **3** and **4** (600 MHz, acquired in ^a^ CDCl_3_ or ^b^ CD_3_OD).

Position	3 ^a^		4 ^a^	4 ^b^	
	*(δ*_C_, Type)	*δ*_H_, Mult. (*J* in Hz)	*δ*_H_, Mult. (*J* in Hz)	*δ*_H_, Mult. (*J* in Hz)	*(δ*_C_, Type)
1	151.5, CH	7.82 s	4.51 d (12.4) 3.84 d (12.4)	4.48 d (12.4) 3.84 d (12.4)	70.8, CH_2_
3	153.5, C				161.8, C
4	114.0, C				104.3, C
4a	143.7, C				152.3, C
5	106.3, CH	5.56 s	5.92 s	5.87 s	116.0, CH
6	200.5, C				201.9, C
7	61.5, C				61.5, C
8	199.5, C				207.4, C
8a	113.5, C				67.2, C
9	17.7, CH3	2.22 s	2.08 s	2.08 s	18.7, CH_3_
10	12.6, CH3	1.89 s	1.83 s	1.87 s	12.9, CH_3_
11	20.6, CH3	1.28 s	1.26 s	1.19 s	17.4, CH_3_
12	31.3, CH2	1.86 q (7.0, 2)	2.25 dq (14.7, 7.4) 1.86 dq (14.7, 7.4)	2.27, dq (14.7, 7.4) 1.80, dq (14.7, 7.4)	33.9, CH_2_
13	9.7, CH3	0.78 t (7.0)	0.89 t (7.4)	0.88, t (7.4)	9.1, CH_3_

**Table 4:** Anti-phytopathogenic activity (IC_50_ in µg/mL) of 2–5 against susceptible phytopathogens. Reference compound: chloramphenicol for *X. campestris* and *P. syringae*; cycloheximide for *P. infestans*.

Phytopathogens	Compound				
	2	3	4	5	Reference
*P. syringae*	>100	>100	>100	92.6	0.7
*X. campestris*	>100	>100	>100	0.9	0.5
*P. infestans*	13.9	>100	>100	1.7	0.3

## Supplementary Materials

The following are available online at https://www.mdpi.com/1660-3397/18/2/73/s1, Figure S1: Phylogenetic tree for taxonomic assignment of isolate S1DA-Helotiales sp. based on sequencing of the ITS 1-5.8S rRNA gene-ITS2 fragment, Figure S2: MN of mono- and co-culture extracts of Helotiales sp., *P. influorescens* sp. and their co-culture with their base peak chromatograms (BPC), Figure S3: MN of mono-culture extracts of *P. nobilis* sp., *Lentithecium* sp. and their co-culture with BPC, Figure S4: MN of mono-culture extracts of *P. influorescens*, *Lentithecium* sp. and their co-culture with BPC, Figure S5: MN of mono-culture extracts of Helotiales sp., *P. nobilis* and their co-culture with BPC, Figure S6: MN of mono-culture extracts of Helotiales sp., *Lentithecium* sp. and their co-culture with BPC, Figure S7: ^1^H NMR spectrum of compound **1** (CDCl_3_, 600 MHz), Figure S8: ^13^C NMR spectrum of compound **1** (CDCl_3_, 150 MHz), Figure S9: DEPT-135 spectrum of compound **1** (CDCl_3_, 150 MHz), Figure S10: HR-ESIMS spectrum of compound **1**, Figure S11: ^1^H NMR spectrum of compound **2** (CD_3_OD, 600 MHz), Figure S12: ^13^C NMR spectrum of compound **2** (CD_3_OD, 150 MHz), Figure S13: COSY spectrum of compound **2** (CD_3_OD, 600 MHz), Figure S14: HSQC spectrum of compound **2** (CD_3_OD, 600 MHz), Figure S15: HMBC spectrum of compound **2** (CD_3_OD, 600 MHz), Figure S16: NOESY spectrum of compound **2**, (CD_3_OD, 600 MHz), Figure S17: ^1^H NMR spectrum of compound **2** (CDCl_3_, 600 MHz), Figure S18: HR-ESIMS spectrum of compound **2**, Figure S19: FT-IR spectrum of compound **2**, Figure S20: ^1^H NMR spectrum of compound **3** (CDCl_3_, 600 MHz), Figure S21: COSY spectrum of compound **3** (CDCl_3_, 600 MHz), Figure S22: HSQC spectrum of compound **3** (CDCl_3_, 600 MHz), Figure S23: HMBC spectrum of compound **3** (CDCl_3_, 600 MHz), Figure S24: NOESY spectrum of compound **3** (CDCl_3_, 600 MHz), Figure S25: HR-ESIMS spectrum of compound **3**, Figure S26: ^1^H NMR spectrum of compound **4** (CD_3_OD, 600 MHz), Figure S27: ^13^C NMR spectrum of compound **4** (CD_3_OD, 150 MHz), Figure S28: COSY spectrum of compound **4** (CD_3_OD, 600 MHz), Figure S29: HSQC spectrum of compound **4** (CD_3_OD, 600 MHz), Figure S30: HMBC spectrum of compound **4** (CD_3_OD, 600 MHz), Figure S31: NOESY spectrum of compound **4** (CD_3_OD, 600 MHz), Figure S32: ^1^H NMR spectrum of compound **4** (CDCl_3_, 600 MHz), Figure S33: HR-ESIMS spectrum of compound **4**, Figure S34: FT-IR spectrum of compound **4**, Figure S35: ^1^H NMR spectrum of compound **5** (CDCl_3_, 600 MHz), Figure S36: ^13^C NMR spectrum of compound **5** (CDCl_3_, 150 MHz), Figure S37: DEPT-135 spectrum of compound **5** (CDCl_3_, 150 MHz), Figure S38: HR-ESIMS spectrum of compound **5**, Table S1: Identification of 8 sediment-derived fungal strains isolated from Windebyer Noor (Nov, 2015) according to the two new BLAST searches. Table S2: In vitro anti-phytopathogenic activity (IC_50_ values µg/mL) of the DCM subextract (D) and fractions obtained therefrom (D1–D11) by elution on a C18 SPE cartridge.
