# Supplementary material for: Design of Fungal Co-Cultivation Based on Comparative Metabolomics and Bioactivity for Discovery of Marine Fungal Agrochemicals
Source: Mar Drugs. 2020 Jan 23;18(2):73. doi: 10.3390/md18020073 (PMC7073616; doi:10.3390/md18020073)

SUPPLEMENTARY MATERIALS

Design of fungal co-cultivation based on comparative metabolomics and bioactivity for discovery of marine fungal agrochemicals

Ernest Oppong-Danquah [1], Paulina Budnicka [1], Martina Blümel [1] and Deniz Tasdemir [1, 2*]

[1] GEOMAR Centre for Marine Biotechnology (GEOMAR-Biotech), Research Unit Marine Natural Products Chemistry, GEOMAR Helmholtz Centre for Ocean Research Kiel, Am Kiel-Kanal 44, 24106 Kiel, Germany; eoppong-danquah@geomar.de (E.O.-D.); pbudnicka@gmail.com (P.B.); mbluemel@geomar.de (M.B.)

[2] Faculty of Mathematics and Natural Science, Kiel University, Christian-Albrechts-Platz 4, 24118 Kiel, Germany

* Correspondence: dtasdemir@geomar.de; Tel.: +49-431-6004430, ORCID ID: 0000-0002-7841-6271

List of Tables **Page No.**

Table S1. Identification of 8 sediment-derived fungal strains isolated from Windebyer Noor (Nov, 2015) according to the two new BLAST searches 4

Table S2. In vitro anti-phytopathogenic activity (IC_{50} values in μg/mL) of the DCM subextract (D) and fractions obtained therefrom (D1–D11) by elution on a C18 SPE cartridge 6

List of Figures

Figure S1. Phylogenetic tree for taxonomic assignment of isolate S1DA-Helotiales sp. based on sequencing of the ITS 1-5.8S rRNA gene-ITS2 fragment. 7

Figure S2. MN of mono-culture extracts of Helotiales sp. (red), *P. influorescens* sp. (green) and their co-culture (blue) with base peak chromatograms (BPC) 8

Figure S3. MN of mono-culture extracts of *P. nobilis* sp. (red), *Lentithecium* sp. (green) and their co-culture (blue) with BPC. 9

Figure S4. MN of mono-culture extracts of *P. influorescens* (red), *Lentithecium* sp. (green) and their co-culture (blue) with BPC. 10

Figure S5. MN of mono-culture extracts of Helotiales sp. (red), *P. nobilis*. (green) and their co-culture (blue) with BPC. 11

Figure S6. MN of mono-culture extracts of Helotiales sp. (red), *Lentithecium* sp. (green) and their co-culture (blue) with BPC. 12

Figure S7. ^{1}H NMR spectrum of compound **1** ($CDCl_3$, 600 MHz). 13

Figure S8. ^{13}C NMR spectrum of compound **1** ($CDCl_3$, 150 MHz). 13

Figure S9. DEPT-135 spectrum of compound **1** ($CDCl_3$, 150 MHz). 14

Figure S10. HR-ESIMS spectrum of compound **1.** 14

Figure S11. ^{1}H NMR spectrum of compound **2** (CD_3OD, 600 MHz). 15

Figure S12. ^{13}C NMR spectrum of compound **2** (CD_3OD, 150 MHz). 15

Figure S13. COSY spectrum of compound **2** (CD_3OD, 600 MHz). 16

Figure S14. HSQC spectrum of compound **2** (CD_3OD, 600 MHz). 16

Figure S15. HMBC spectrum of compound **2** (CD_3OD, 600 MHz). 17

Figure S16. NOESY spectrum of compound **2**, (CD_3OD, 600 MHz). 17

Figure S17. ^{1}H NMR spectrum of compound **2** ($CDCl_3$, 600 MHz). 18

Figure S18. HR-ESIMS spectrum of compound **2.** 18

Figure S19. FT-IR spectrum of compound **2.** 18

Figure S20. ^{1}H NMR spectrum of compound **3** ($CDCl_3$, 600 MHz). 19

Figure S21. COSY spectrum of compound **3** ($CDCl_3$, 600 MHz). 19

Figure S22. HSQC spectrum of compound **3** ($CDCl_3$, 600 MHz). 20

Figure S23. HMBC spectrum of compound **3** ($CDCl_3$, 600 MHz). 20

Figure S24. NOESY spectrum of compound **3** ($CDCl_3$, 600 MHz). 21

Figure S25. HR-ESIMS spectrum of compound **3.** 21

Figure S26. ^{1}H NMR spectrum of compound **4** (CD_3OD, 600 MHz). 22

Figure S27. ^{13}C NMR spectrum of compound **4** (CD_3OD, 150 MHz). 22

Figure S28. COSY spectrum of compound **4** (CD_3OD, 600 MHz). 23

Figure S29. HSQC spectrum of compound **4** (CD_3OD, 600 MHz). 23

Figure S30. HMBC spectrum of compound **4** (CD_3OD, 600 MHz). 24

Figure S31. NOESY spectrum of compound **4** (CD_3OD, 600 MHz). 24

Figure S32. ^{1}H NMR spectrum of compound **4** ($CDCl_3$, 600 MHz). 25

Figure S33. HR-ESIMS spectrum of compound **4.** 25

Figure S34. FT-IR spectrum of compound **4.** 25

Figure S35. ^{1}H NMR spectrum of compound **5** ($CDCl_3$, 600 MHz). 26

Figure S36. ^{13}C NMR spectrum of compound **5** ($CDCl_3$, 150 MHz). 26

Figure S37. DEPT-135 spectrum of compound **5** ($CDCl_3$, 150 MHz). 27

Figure S38. HR-ESIMS spectrum of compound **5.** 27

Table S1. Identification of 8 sediment-derived fungal strains isolated from Windebyer Noor (Nov., 2015) according to the two new BLAST searches (13-08-2019). Self-hits are excluded from the similarity table. BLAST-ALL: shows results from nucleotide BLAST against highly similar sequences. BLAST-TYPE: shows results from nucleotide BLAST against highly similar sequences only from type material. Acc. No. is accession number.

Acc. No. of strains used	BLAST-ALL			BLAST-TYPE			Taxonomic assignment
	Acc. No.	Name	% similarity	Acc. No.	Name	% similarity	
MH791233	JF740228.1	*Plenodomus influorescens* strain CBS 143.84	100	NR_111619.1	*Plenodomus enteroleucus* CBS 142.84 TYPE	96.23	*Plenodomus influorescens*
	MK495988.1	*Plenodomus lindquistii* voucher MF-Ha16-005	97.74	NR_119957.1	*Plenodomus visci* TYPE	95.88	
	MK495987.1	*Plenodomus lindquistii* voucher MF-Ha16-004	97.74	MH858430.1	*Plenodomus congestus* strain CBS 244.64 TYPE	95.49	
	MK495986.1	*Plenodomus lindquistii* voucher MF-Ha16-001	97.74	NR_111068.1			
				NR_153896.1	*Comoclathris spartii MFLUCC 13-0214 TYPE*	95.09	
MH791253	MN105533.1	*Penicillium* sp. isolate SL71_37c_D	100	MH854996.1	*Penicillium bialowiezense* strain CBS 227.28 TYPE	100	*Penicillium bialowiezense*
				NR_111323.1			
	MN105477.1	*Penicillium* sp. isolate SL16_77a_G2	100	KC411734.1	*Penicillium brunneostoloniferum* strain CBS 317.59	98.02	
	MN105342.1	*Penicillium* sp. isolate SL63_46_G	100	NR_121299.1	*Penicillium brevicompactum* NRRL 2011	98.02	
	MN105326.1	*Penicillium* sp. isolate SL61_6	100	KF465776.1	*Penicillium brevicompactum* strain CBS 257.29	98.02	
MH791254	MK907754.1	*Sarocladium strictum isolate Z210A*	99.33	GQ376096.2	*Sarocladium strictum isolate CBS 346.70 TYPE*	99.33	*Sarocladium strictum*
				NR_111145.1			
	LC433837.1	*Sarocladium sp. MAFF307188*	99.33	AY138845.1			
				AY566998.1			
	MK299148.1	*Acremonium sp. (in: Hypocreales) isolate LWU_50*	99.33	FN691453.1	*Acremonium strictum nucleomorph CBS:346.70*	99.33	
				MH859409.1	*Sarocladium bactrocephalum strain CBS 749.69*	97.99	
	MH268081.1	*Sarocladium sp. strain AHB01_8B*	99.33	NR_145044.1			
				NR_145046.1	*Sarocladium pseudostrictum UTHSC 02-1892*	97.09	
MH791292	MK796144.1	*Neocucurbitaria cava* isolate cp75.3	100	NR_160112.1	*Neocucurbitaria cava* CBS 257.68 TYPE	99.73	*Neocucurbitaria sp.*
	MK460389.1	*Neocucurbitaria* sp. strain EXF-12877	100	JF740260.1			
	MK460388.1	*Neocucurbitaria* sp. strain EXF-12880	100	NR_156358.1	*Neocucurbitaria juglandicola* CBS 142390 TYPE	99.73	
	MK460387.1	*Neocucurbitaria* sp. strain EXF-12446	100	MF795773.1	*Neocucurbitaria juglandicola* strain BW6	99.73	
				NR_156359.1	*Neocucurbitaria populi* CBS 142393 TYPE	99.47	
MH791174	MH791174.1	*Pyrenochaeta nobilis* isolate SICB	100	NG_062727.1	*Pyrenochaeta nobilis* CBS 407.76 TYPE	98.38	*Pyrenochaeta nobilis*
	NG_062727.1	*Pyrenochaeta nobilis* CBS 407.76 TYPE	98.38	MF795792.1			
	MF795792.1			DQ898287.1			
	DQ898287.1			MF795794.1	*Seltsamia ulmi* strain L150	98.24	
	EU710832.1	*Pyrenochaeta* sp. 14009	98.31	NG_062728.1	*Pseudopyrenochaeta lycopersici* CBS 306.65 TYPE	98.11	

	U42481.1	*Cucurbitaria berberidis*	98.31	DQ898289.1			
				NG_063079.1	*Septoriella leuchtmannii CBS 459.84 TYPE*	98.04	
MH791258 *	KT923227.1	Fungal sp. strain OTU53	99.79	MH862690.1	*Helicodendron microsporum* strain CBS 100149 TYPE	91.50	Helotiales sp.
				NR_137974.1			
	JX507714.1	Helotiales sp. 203 OA-2013	98.90	MH862609.1	*Helicodendron websteri* strain CBS 745.96	91.50	
				MH857844.1	*Dimorphospora foliicola* strain CBS 221.59 TYPE	91.24	
	JX507688.1	Helotiales sp. 104 OA-2013	98.89	NR_153969.1			
				DQ202518.1			
	JX507683.1	Helotiales sp. 96 OA-2013	98.89	NR_160181.1	*Hydrocina chaetocladia* CBS 249.90	87.41	
				MH862207.1			
MH791244 **	MH791287.1	*Phoma* sp. isolate 41RWS2	99.71	NR_154108.1	*Lentithecium pseudoclioninum* HHUF 29055 TYPE	93.64	*Lentithecium* sp.**
	MH791275.1	*Phoma* sp. isolate 8ES2	99.71	AB809633.1	*Lentithecium pseudoclioninum* strain: KT 1113	93.64	
	MH791270.1	*Phoma* sp. isolate 26XWS2	99.71	NR_154137.1	*Lentithecium clioninum* HHUF 28199	93.35	
	MH791242.1	*Phoma* sp. isolate 27XWS2	99.71	LC014566.1	*Lentithecium clioninum* KT1149A	93.35	
MH791275 **	MH791287.1	*Phoma* sp. isolate 41RWS2	100	NR_158534.1	*Lentithecium carbonneanum* CBS 144076 TYPE	95.21	*Lentithecium* sp.**
	MH791273.1	*Phoma* sp. isolate 27XWS2	99.78	MH062991.1	*Lentithecium carbonneanum*	95.21	
	MH791270.1	*Phoma* sp. isolate 26XWS2	99.57	NR_154108.1	*Lentithecium pseudoclioninum* HHUF 29055 TYPE	92.75	
	KU179250.1	Fungal endophyte isolate 15	97.01	AB809633.1	*Lentithecium pseudoclioninum* KT 1113	92.75	

*: Taxonomic assignment was not possible to a higher rank than order level. Phylogenetic tree calculation (Fig. S27) suggests that this isolate may belong to a yet undescribed family within the order Helotiales

**: Re-sequencing the 28S rRNA gene of these isolates (9ES2- MH791244, 8ES2- MH791275) allowed classification as *Lentithecium* sp.

Table S2. In vitro antiphytopathogenic activity (IC_{50} values in µg/mL) of the DCM subextract (D) and fractions obtained therefrom (D1–D11) by elution on a C18 SPE cartridge. Test phytopathogens include Pss, *P. syringae*; Xc, *X. campestris*, Ea, *E. amylovora*; Rs, *R. solanacearum*; Pi, *P. infestans*; Mo. *M. oryzae*. 0.5% DMSO was used as a solvent control. Positive controls for Xc, Ea and Pss: chloramphenicol, for Rs: tetracycline, for Mo: nystatin and for Pi: cycloheximide.

Sample	**Pss**	**Xc**	**Ea**	**Rs**	**Pi**	**Mo**
D	> 100	0.9	> 100	> 100	2.3	> 100
D1/2	> 100	34.1	> 100	> 100	> 100	> 100
D 3	> 100	> 100	> 100	> 100	> 100	> 100
D 4/5	> 100	56.1	> 100	> 100	> 100	> 100
D 6	83.8	8.5	> 100	> 100	15.6	> 100
D 7	18.8	3.1	> 100	> 100	4.3	> 100
D 8	17.2	2.1	> 100	> 100	3,1	> 100
D 9	32.0	3.9	> 100	> 100	3,1	> 100
D 10	> 100	7.1	> 100	> 100	> 100	> 100
D 11	> 100	13.3	> 100	> 100	> 100	> 100
Positive control	0.7	0.5	0.7	1.0	0.3	0.4

Figure S1. Phylogenetic tree for taxonomic assignment of isolate S1DA-Helotiales sp. based on sequencing of the ITS 1-5.8S rRNA gene-ITS2 fragment. Taxonomic affiliation is only possible to order level due to the low similarity (91.5%, Table S1) to related type strains. Closest relatives (98.8-99.8% similarity) of our isolate S1DA are other isolates only identified to order level.

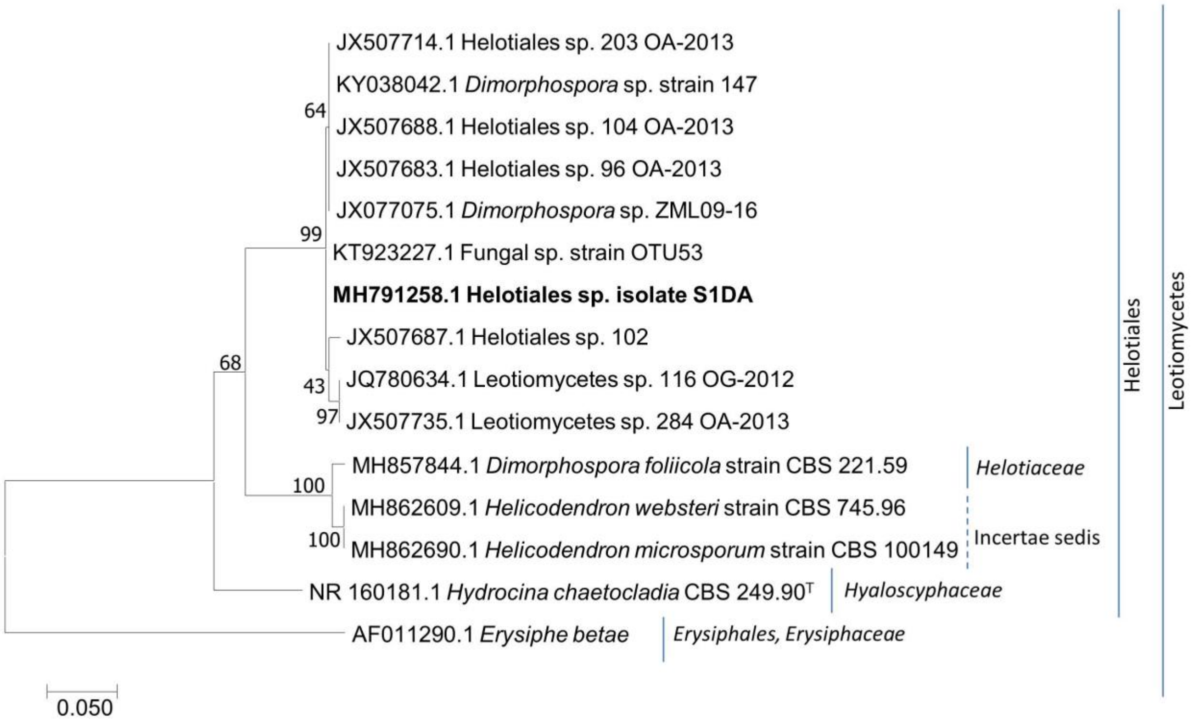

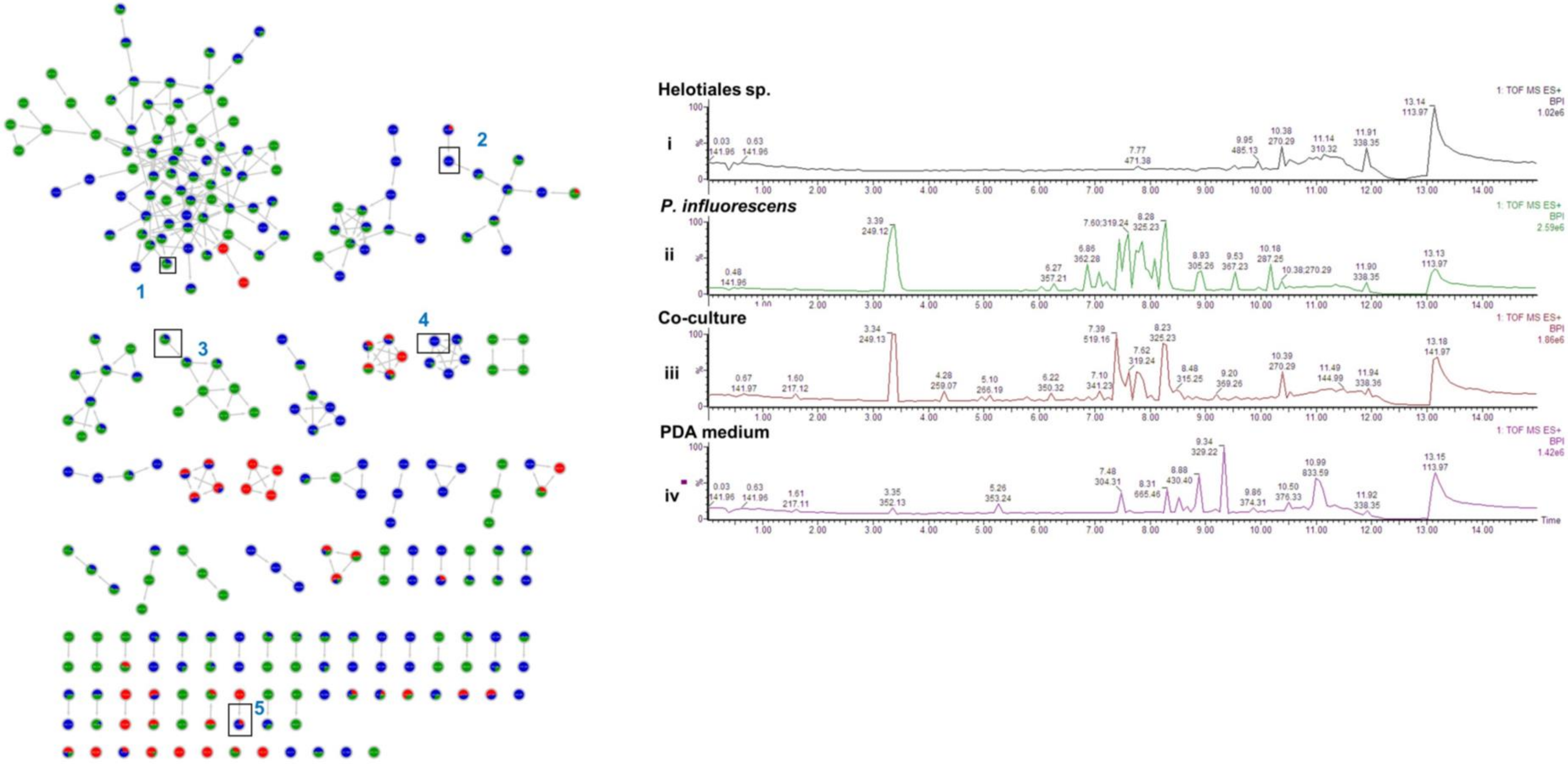

(A) (B)

Figure S2. (**A**) MN of mono-culture extracts of Helotiales sp. (red), *P. influorescens* sp. (green) and their co-culture (blue). Nodes were annotated putatively as: **1**- phomactin B2, **2**- dehydroxybisdethiobis(methylthio)gliotoxin, **3**- (5E)-4-hydroxy-12-methyl-1-oxacyclododec-5-ene-2,8-dione, **4**- cephalochromin, **5**- monodictysin B. (**B**) Base peak chromatograms of Helotiales sp. mono-culture (i), *P. influorescens* sp. mono-culture (ii), their co-culture (iii), blank PDA medium (iv).

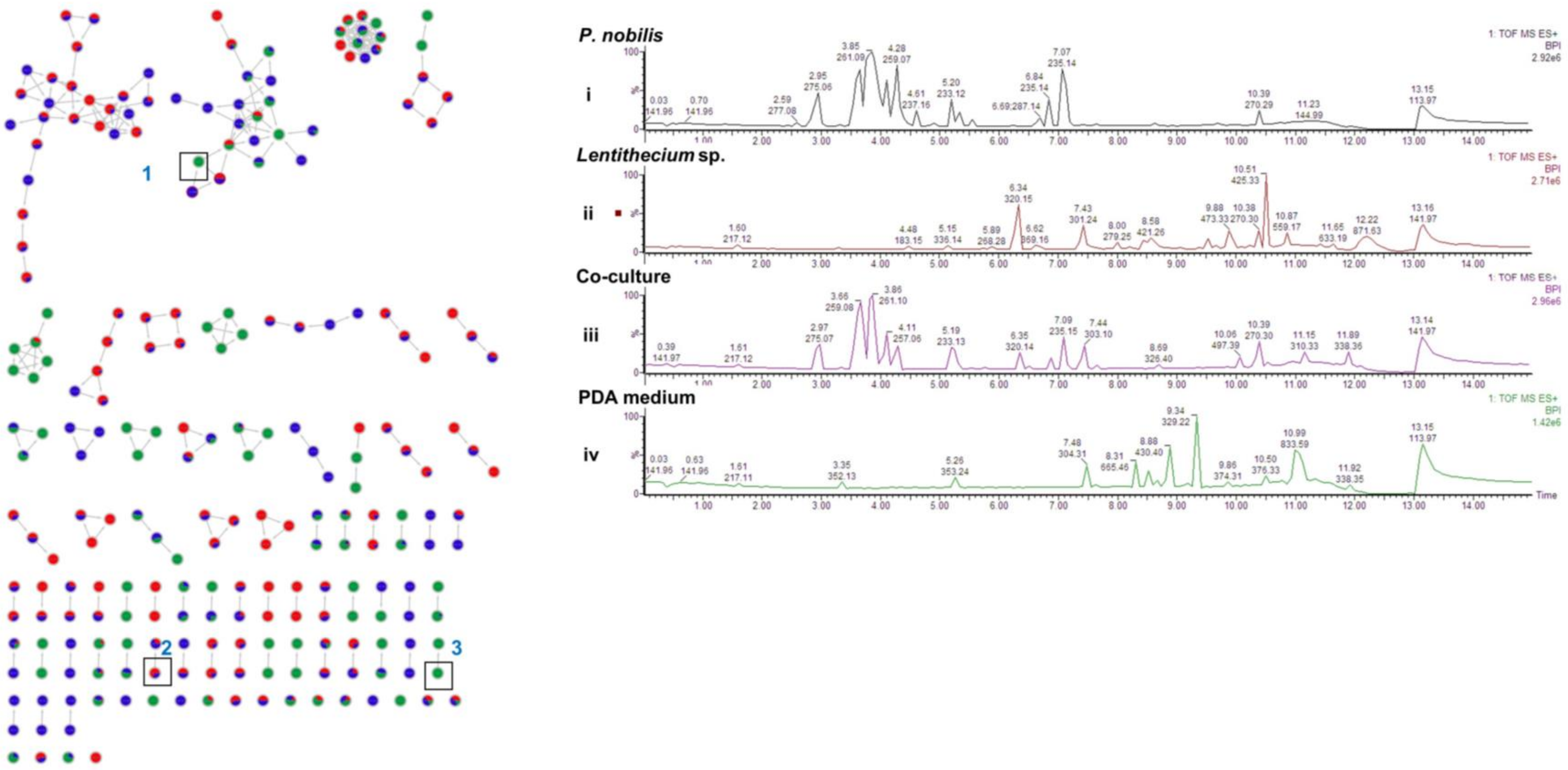

(A) **(B)**

Figure S3. (**A**) MN of mono-culture extracts of *P. nobilis* sp. (red), *Lentithecium* sp. (green) and their co-culture (blue). Nodes were annotated putatively as: **1**- 3-acetyl-5-isopropyl-pyrrolidine-2,4-dione, **2**- spiciferinone, **3**- truncatone. (**B**) Base peak chromatograms of *P. nobilis* mono-culture (i), *Lentithecium* sp. mono-culture (ii), their co-culture (iii), blank PDA medium (iv).

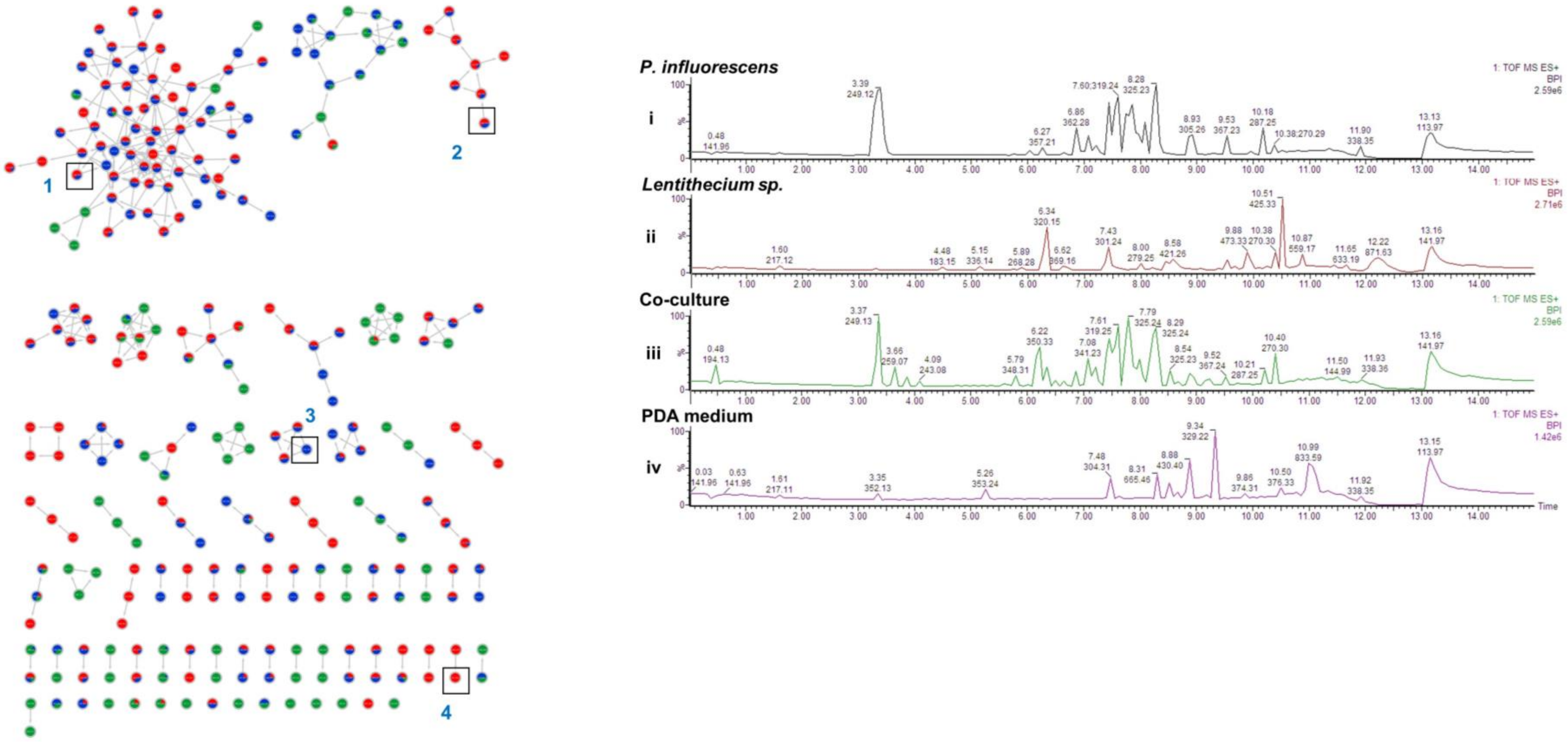

(A) **(B)**

Figure S4. (**A**) MN of mono-culture extracts of *P. influorescens* (red), *Lentithecium* sp. (green) and their co-culture (blue). Nodes were annotated putatively as: **1**- phomactin B2, **2**- (5E)-4-hydroxy-12-methyl-1-oxacyclododec-5-ene-2,8-dione, **3**- cephalochromin, **4**- fumonisin B4. (**B**) Base peak chromatograms of *P. influorescens* mono-culture (i), *Lentithecium* sp. mono-culture (ii), their co-culture (iii), blank PDA medium (iv).

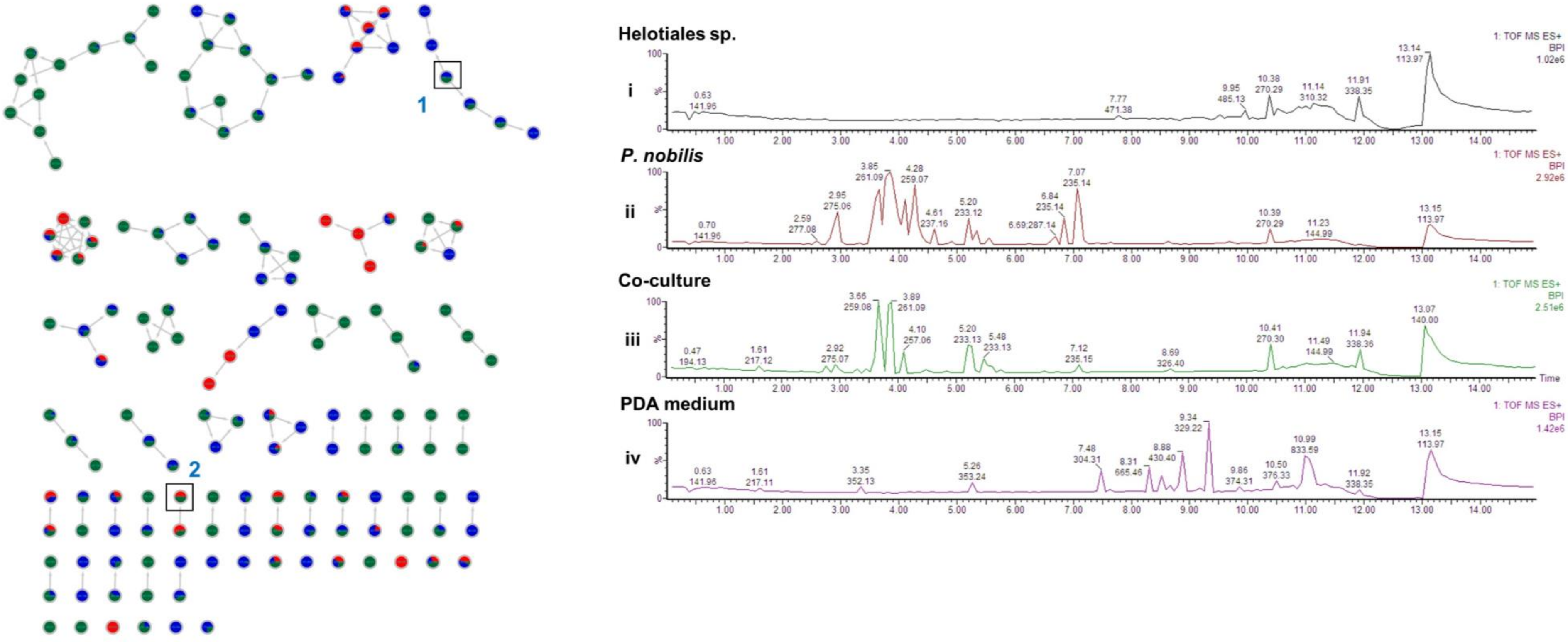

(A) **(B)**

Figure S5. (**A**) MN of mono-culture extracts of Helotiales sp. (red), *P. nobilis*. (green) and their co-culture (blue). Nodes were annotated putatively as: **1**- spiciferinone, **2**- monodictysin B. (**B**) Base peak chromatograms of Helotiales sp. mono-culture (i), *P. nobilis* mono-culture (ii), their co-culture (iii), blank PDA medium (iv).

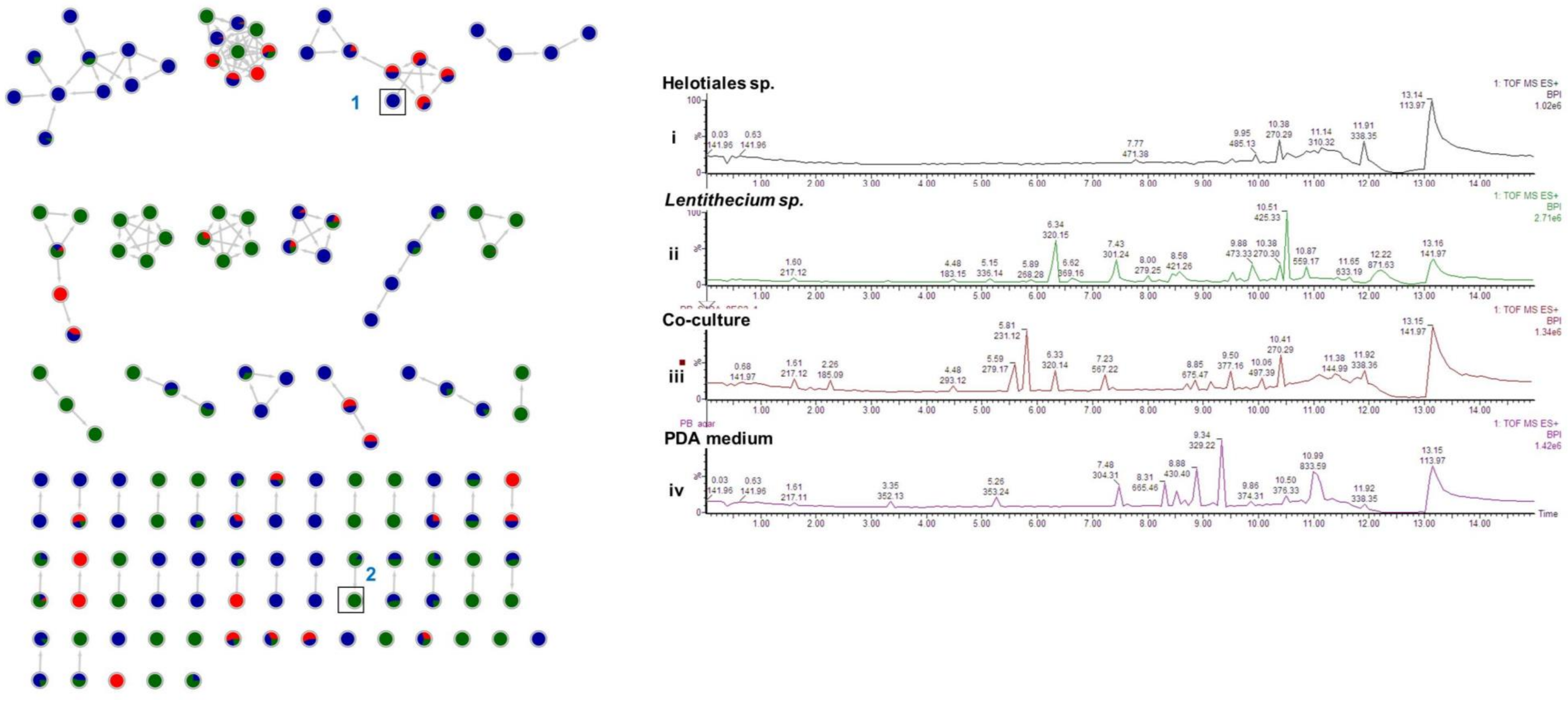

(A) **(B)**

Figure S6. (**A**) MN of mono-culture extracts of Helotiales sp. (red), *Lentithecium* sp. (green) and their co-culture (blue). Nodes were annotated putatively as: **1**- truncatone, **2**- 3-acetyl-5-isopropyl-pyrrolidine-2,4-dione. (**B**) Base peak chromatograms of Helotiales sp. mono-culture (i), *Lentithecium* sp. mono-culture (ii), their co-culture (iii), blank PDA medium (iv).

Figure S7. ^{1}H NMR spectrum of compound **1** ($CDCl_3$, 600 MHz).

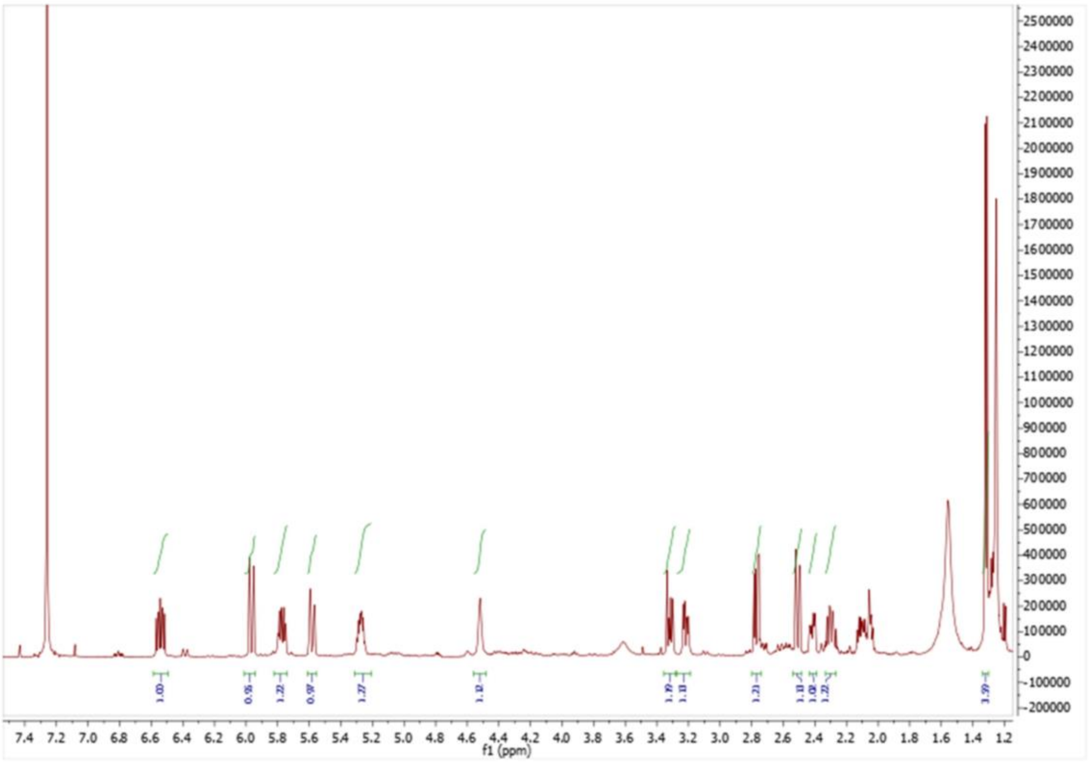

Figure S8. ^{13}C NMR spectrum of compound **1** ($CDCl_3$, 150 MHz).

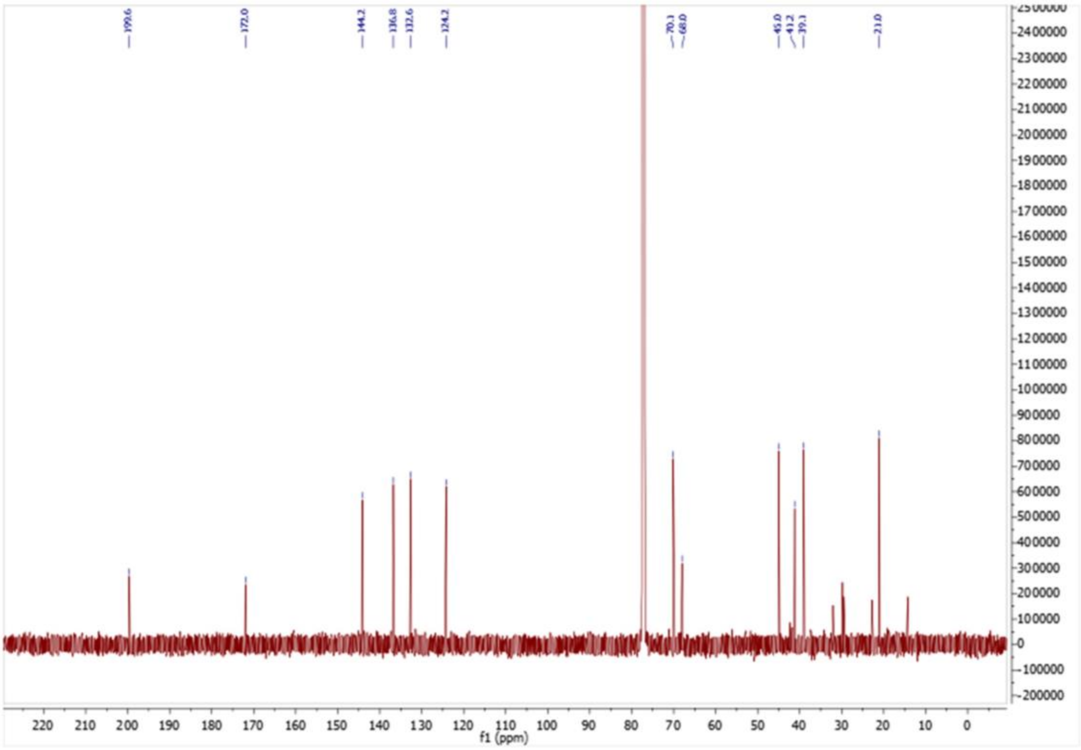

Figure S9. DEPT-135 spectrum of compound **1** ($CDCl_3$, 150 MHz).

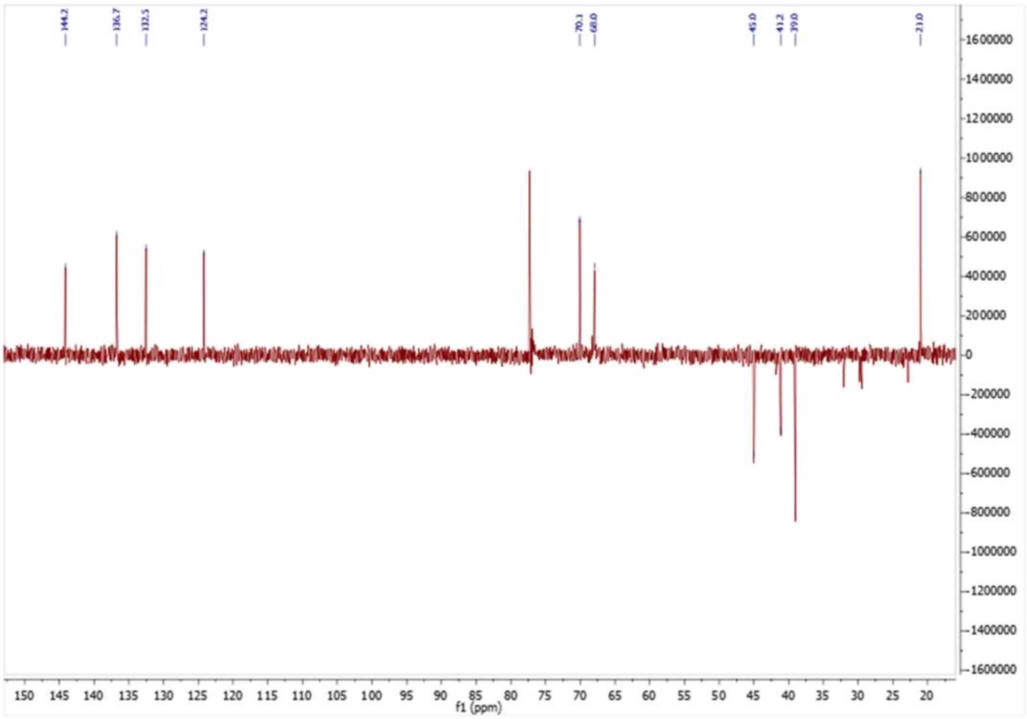

Figure S10. HR-ESIMS spectrum of compound **1.**

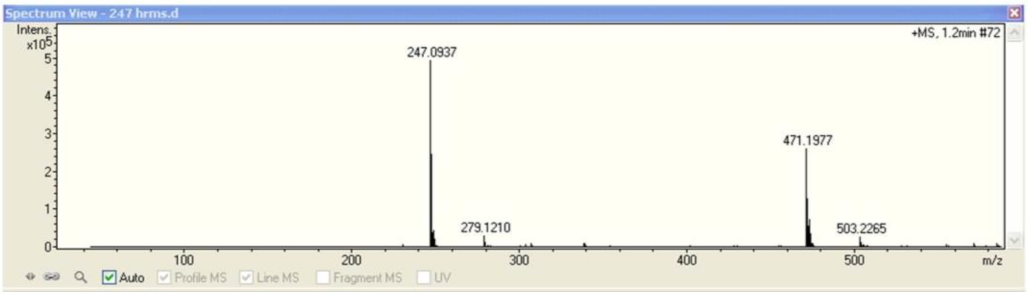

Figure S11. ^{1}H NMR spectrum of compound **2** (CD_3OD, 600 MHz).

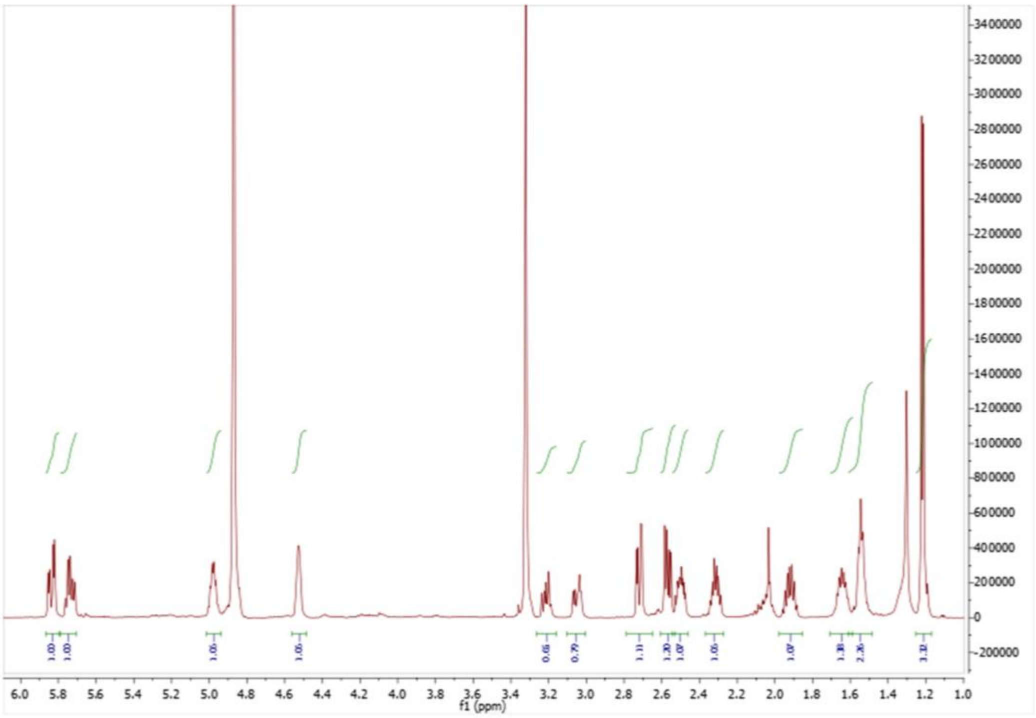

Figure S12. ^{13}C NMR spectrum of compound **2** (CD_3OD, 150 MHz).

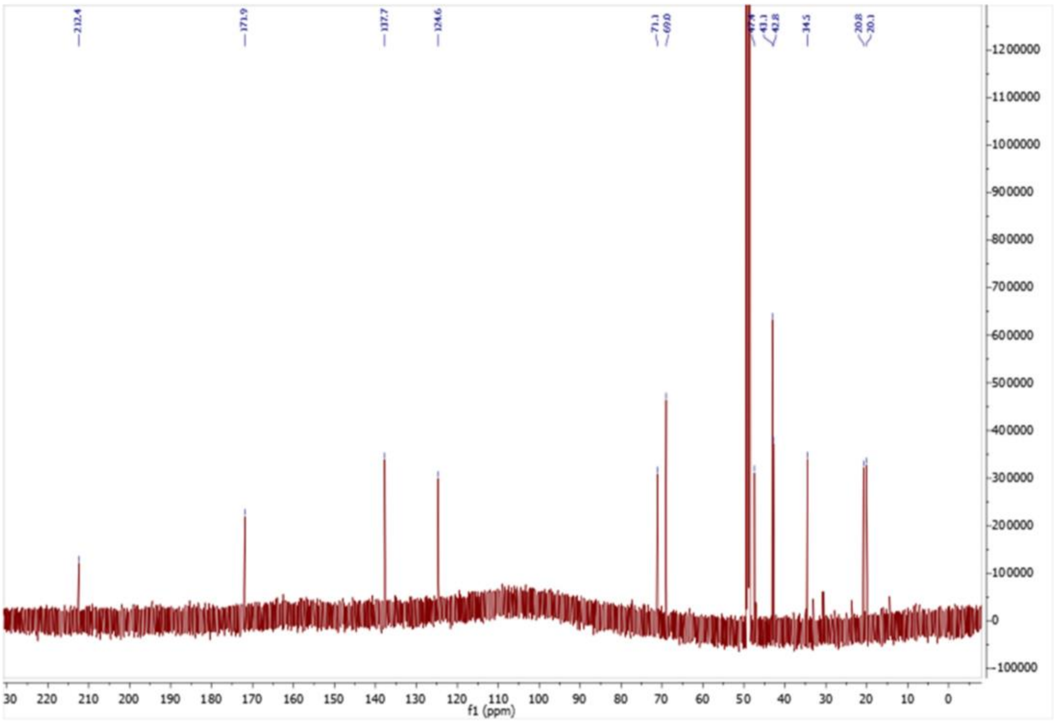

Figure S13. COSY spectrum of compound **2** (CD_3OD, 600 MHz).

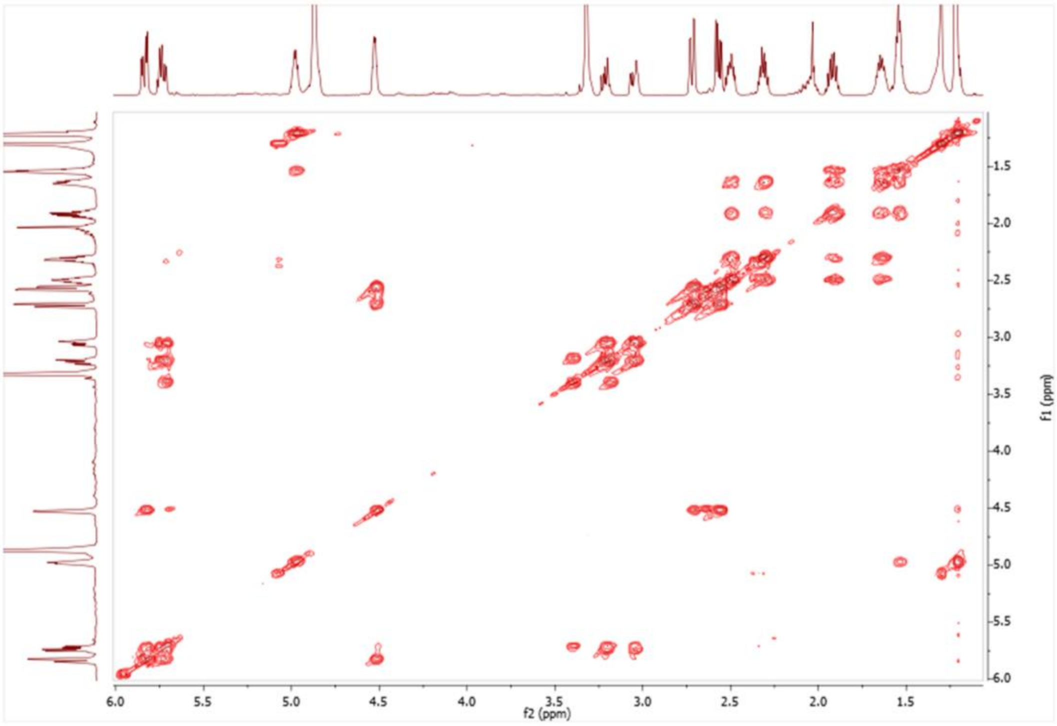

Figure S14. HSQC spectrum of compound **2** (CD_3OD, 600 MHz).

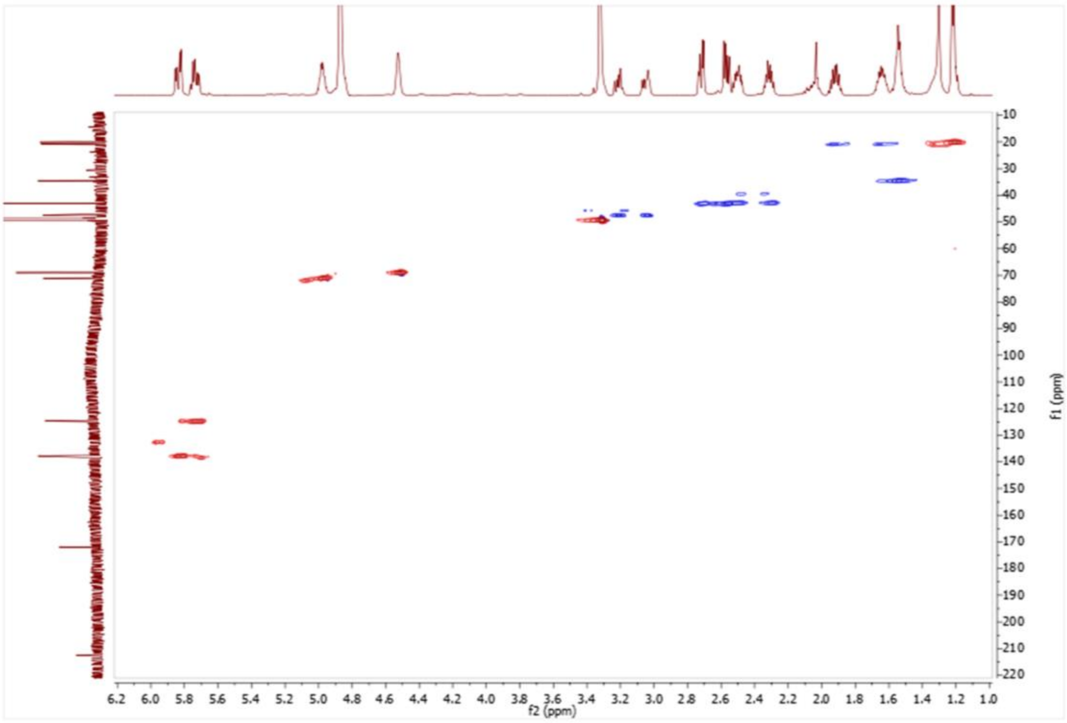

Figure S15. HMBC spectrum of compound **2** (CD_3OD, 600 MHz).

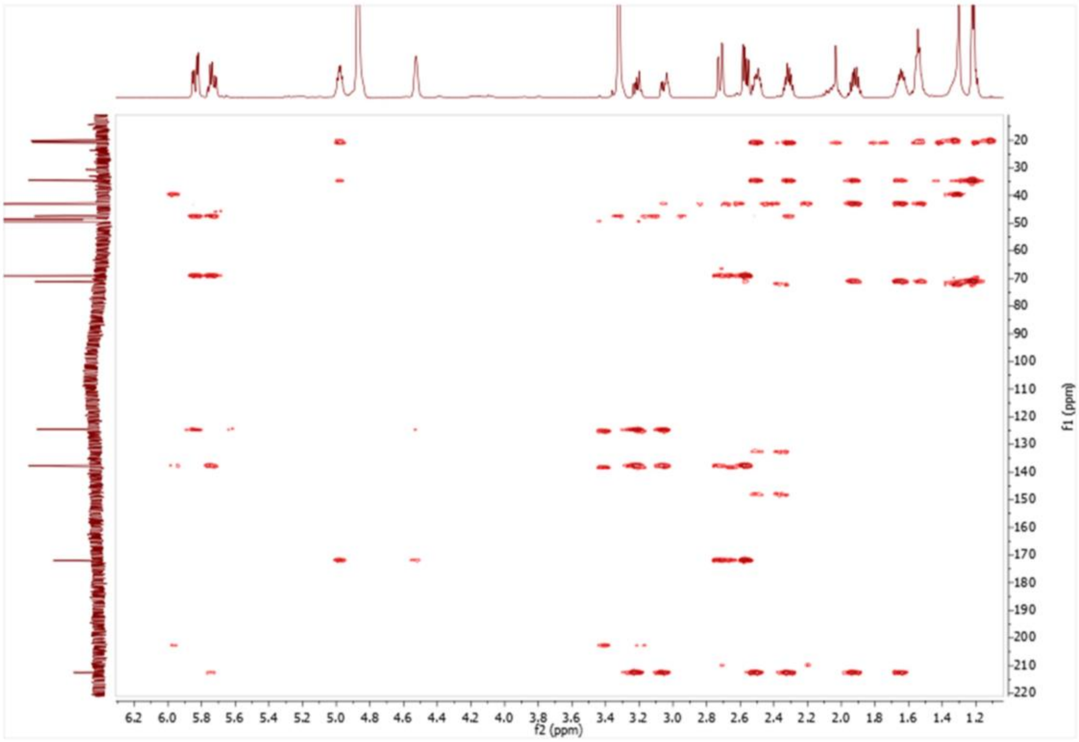

Figure S16. NOESY spectrum of compound **2**, (CD_3OD, 600 MHz).

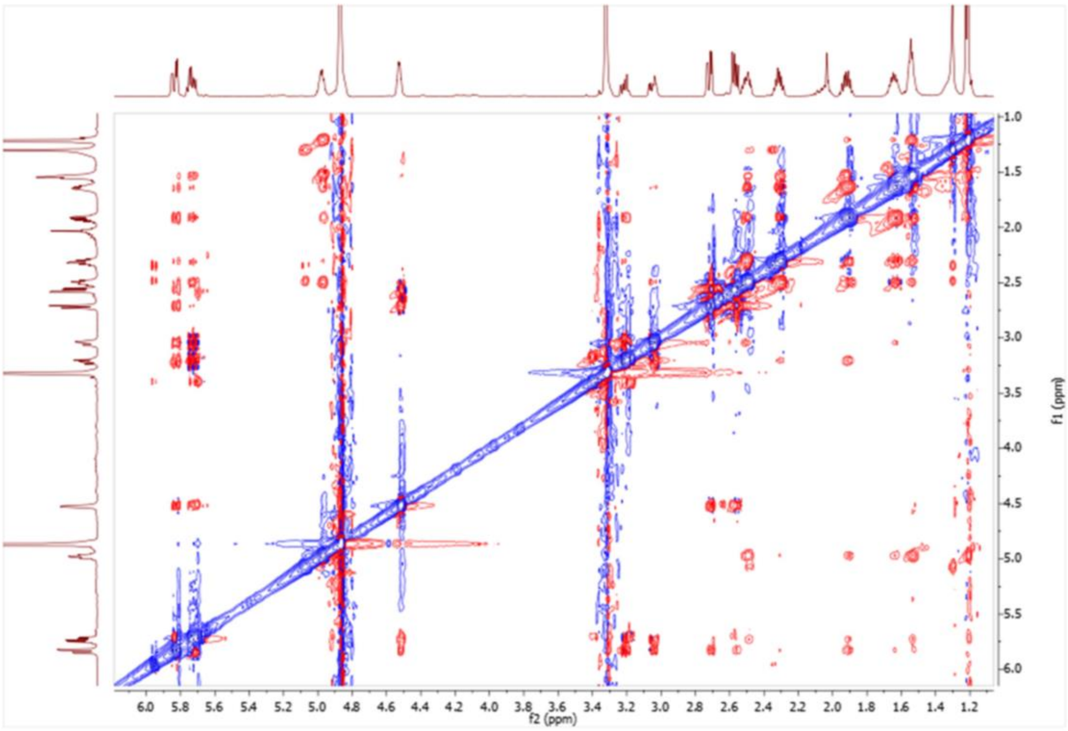

Figure S17. ^{1}H NMR spectrum of compound **2** ($CDCl_3$, 600 MHz).

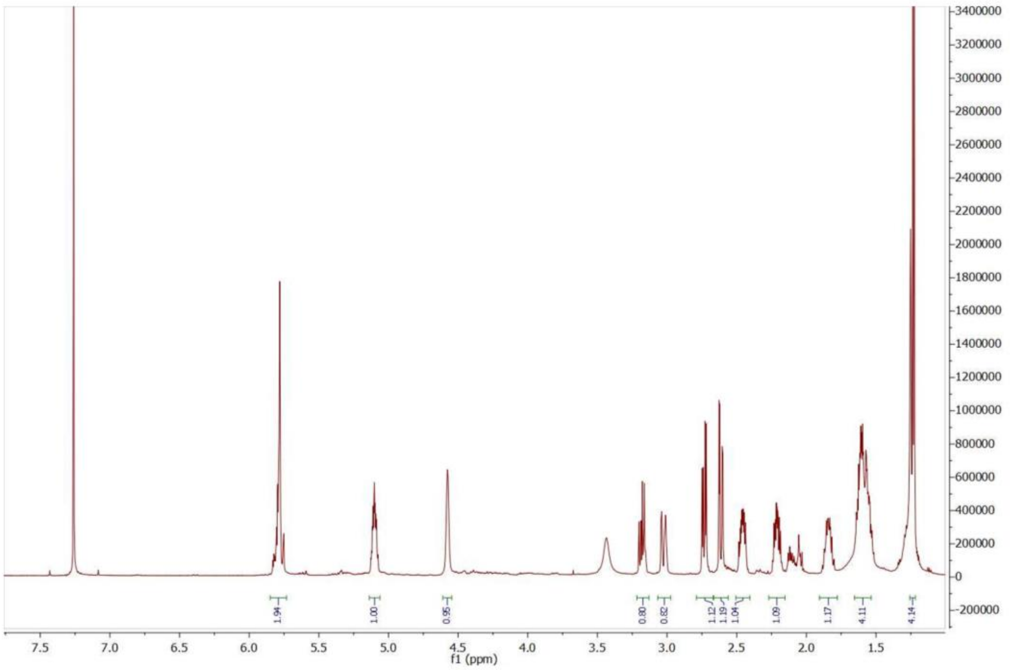

Figure S18. HR-ESIMS spectrum of compound **2.**

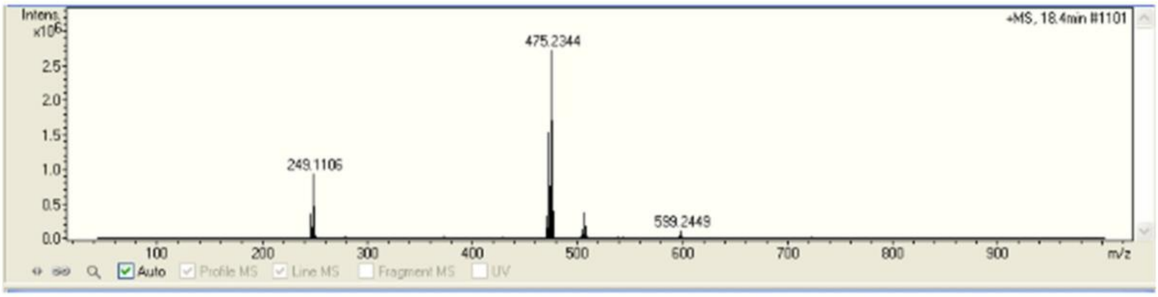

Figure S19. FT-IR spectrum of compound **2.**

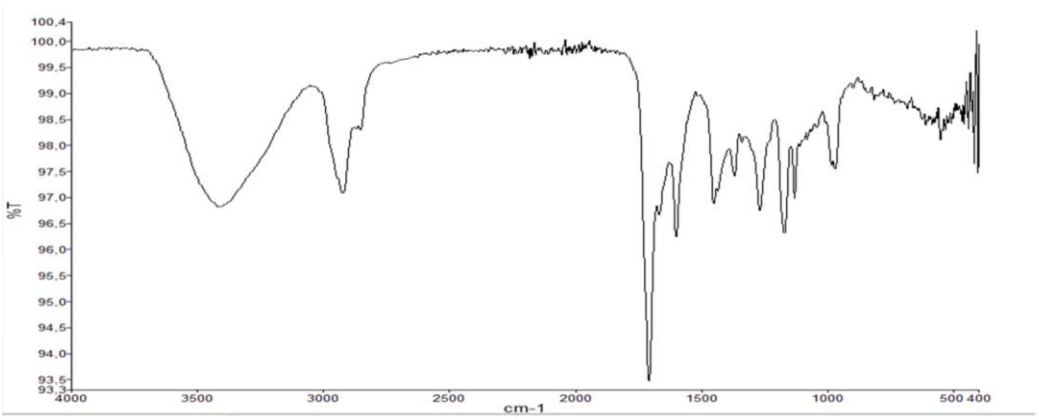

Figure S20. ^{1}H NMR spectrum of compound **3** ($CDCl_3$, 600 MHz).

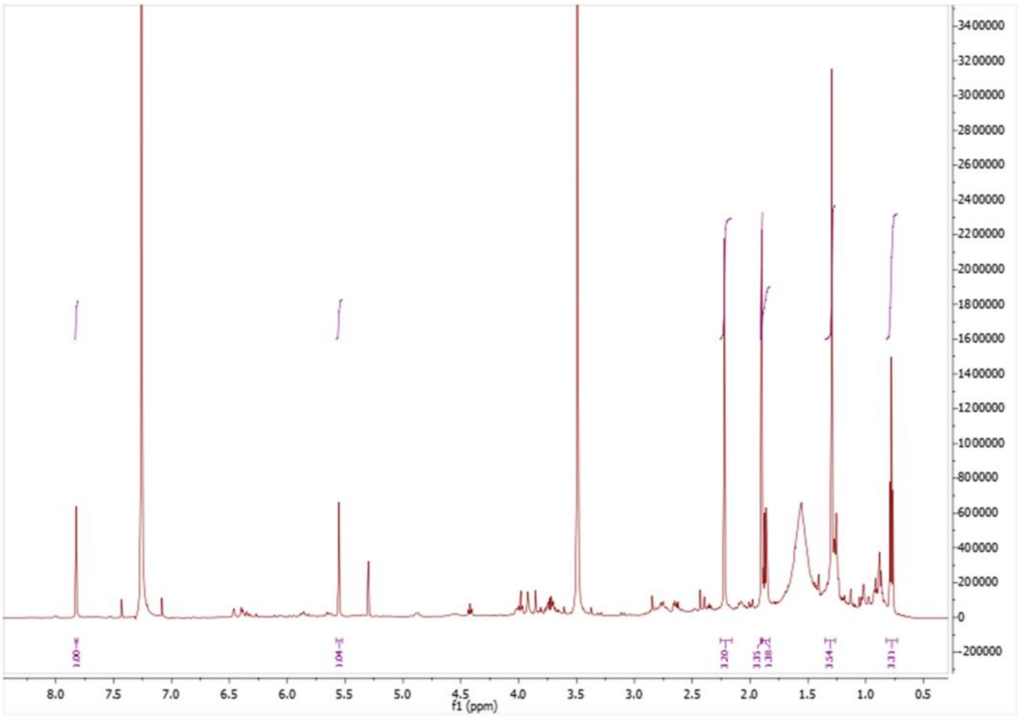

Figure S21. COSY spectrum of compound **3** ($CDCl_3$, 600 MHz).

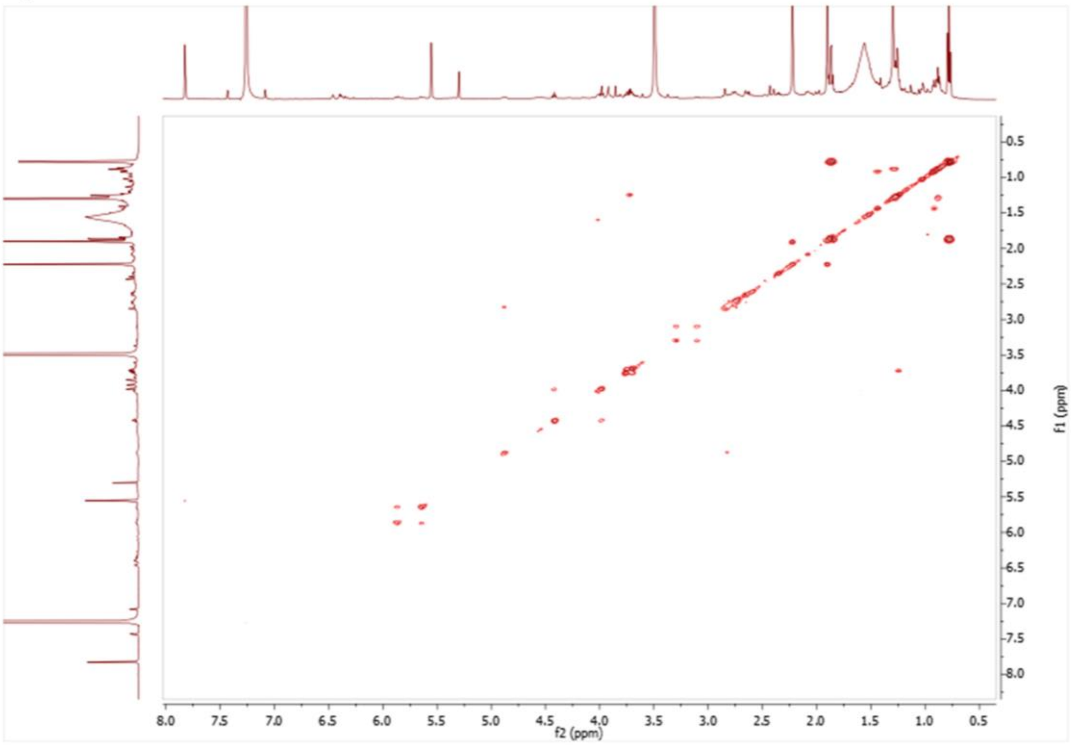

Figure S22. HSQC spectrum of compound **3** ($CDCl_3$, 600 MHz).

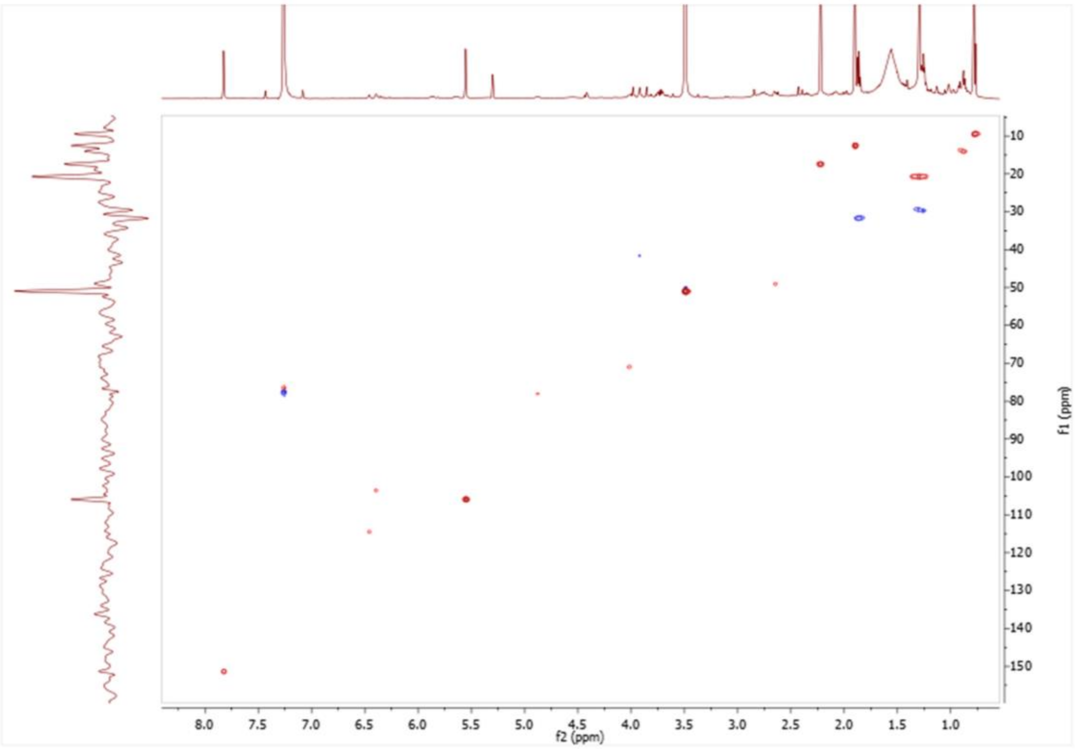

Figure S23. HMBC spectrum of compound **3** ($CDCl_3$, 600 MHz).

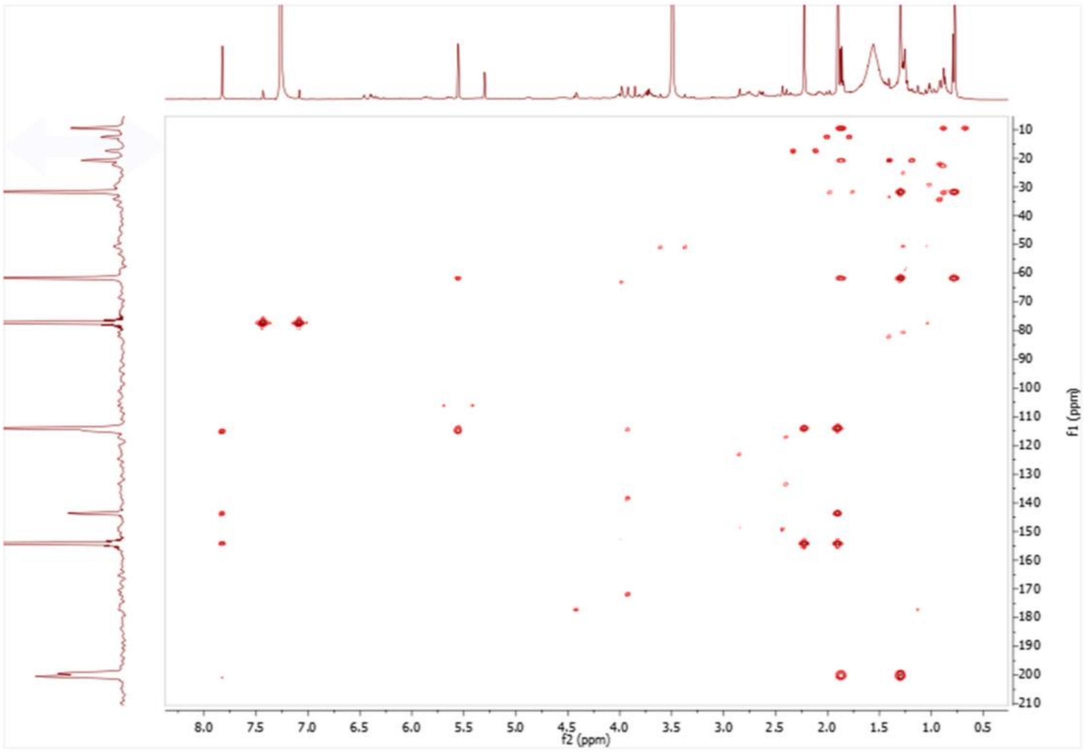

Figure S24. NOESY spectrum of compound **3** ($CDCl_3$, 600 MHz).

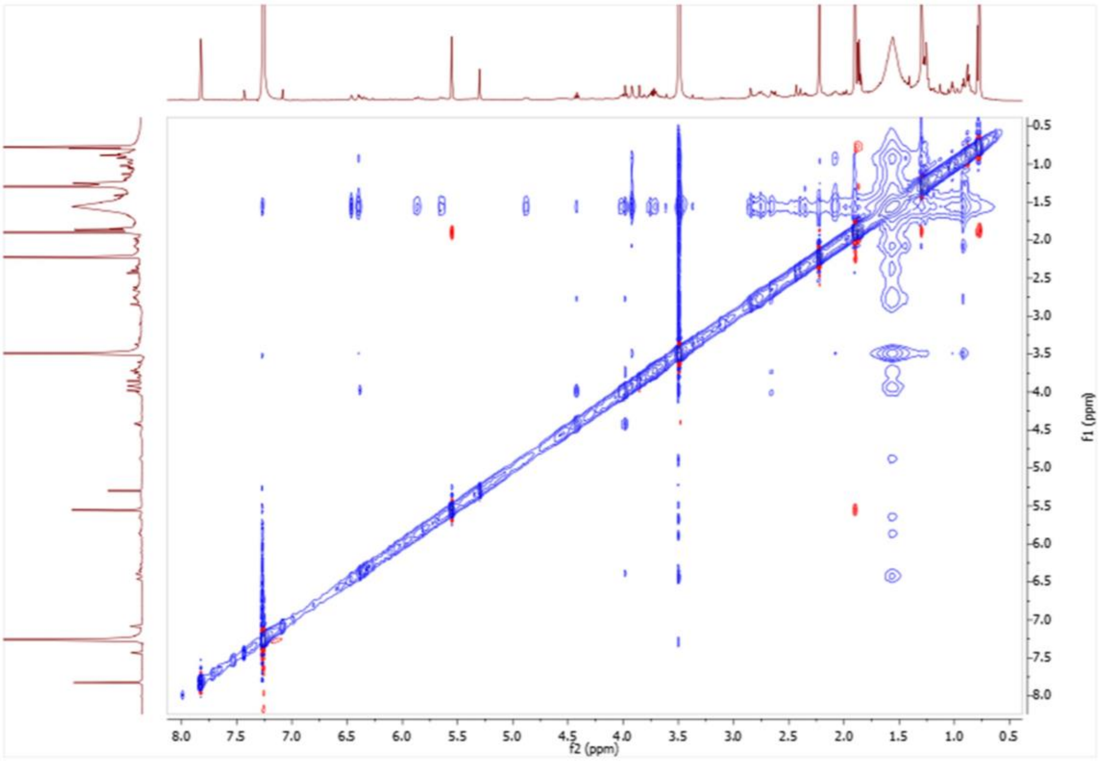

Figure S25. HR-ESIMS spectrum of compound **3.**

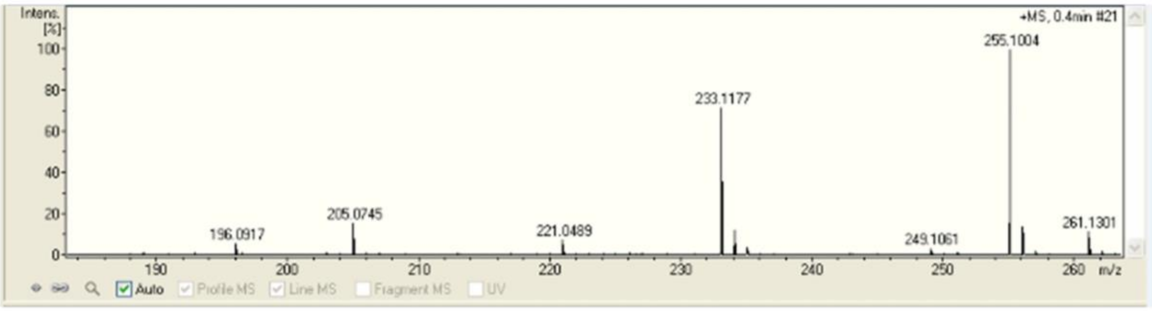

Figure S26. ^{1}H NMR spectrum of compound **4** (CD_3OD, 600 MHz).

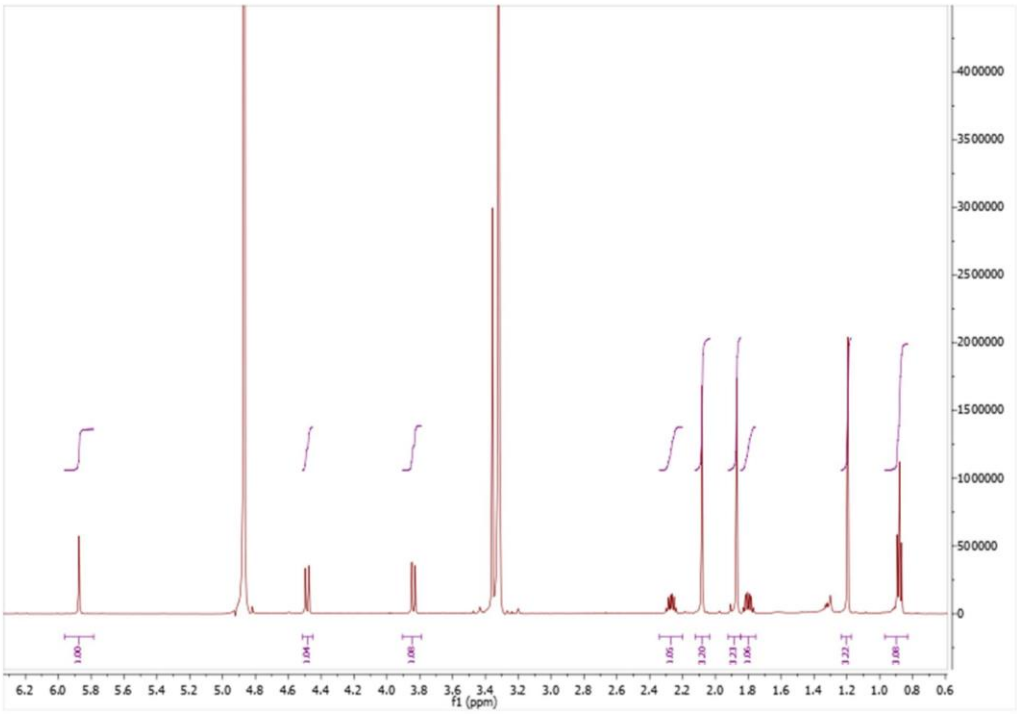

Figure S27. ^{13}C NMR spectrum of compound **4** (CD_3OD, 150 MHz).

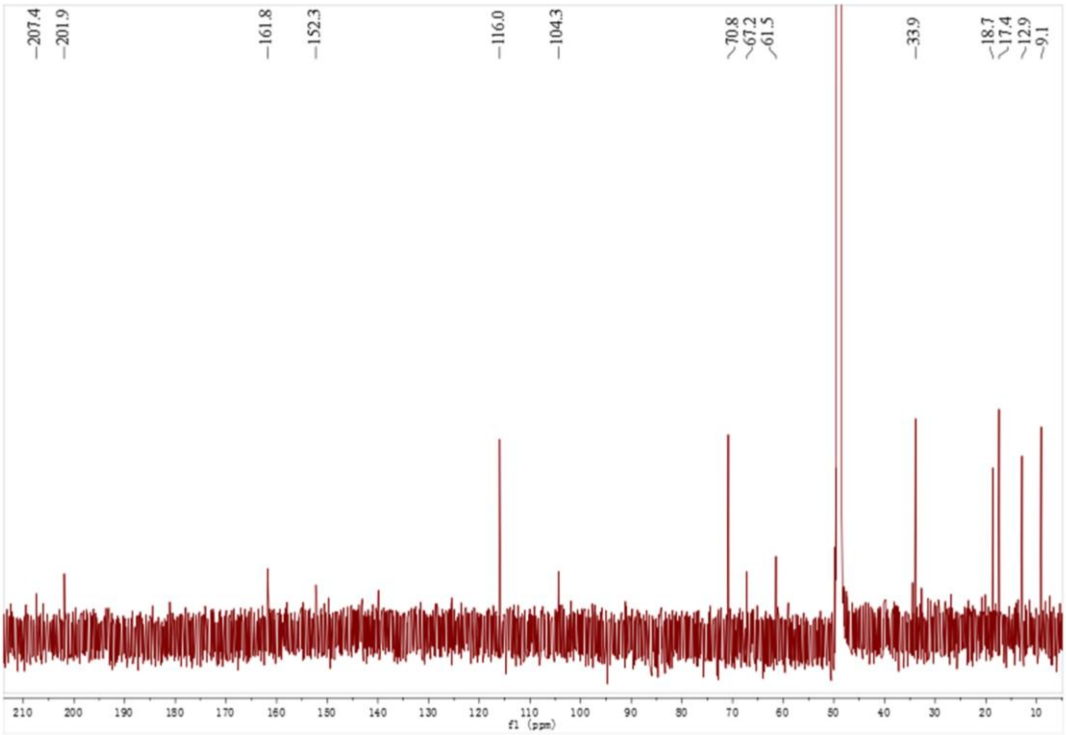

Figure S28. COSY spectrum of compound **4** (CD_3OD, 600 MHz).

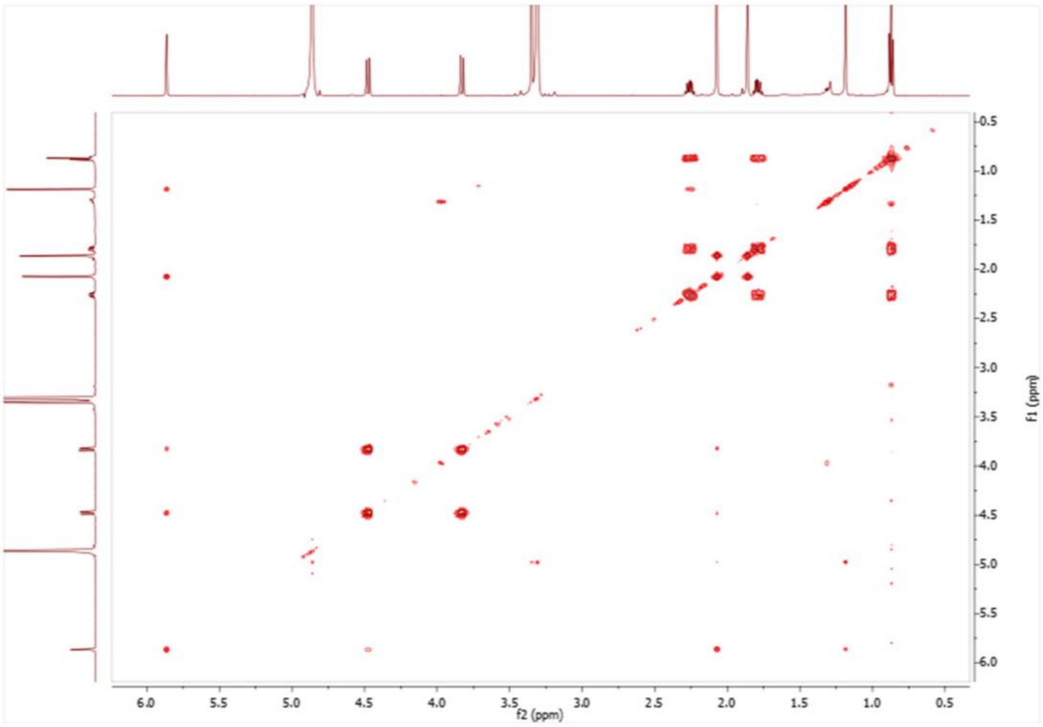

Figure S29. HSQC spectrum of compound **4** (CD_3OD, 600 MHz).

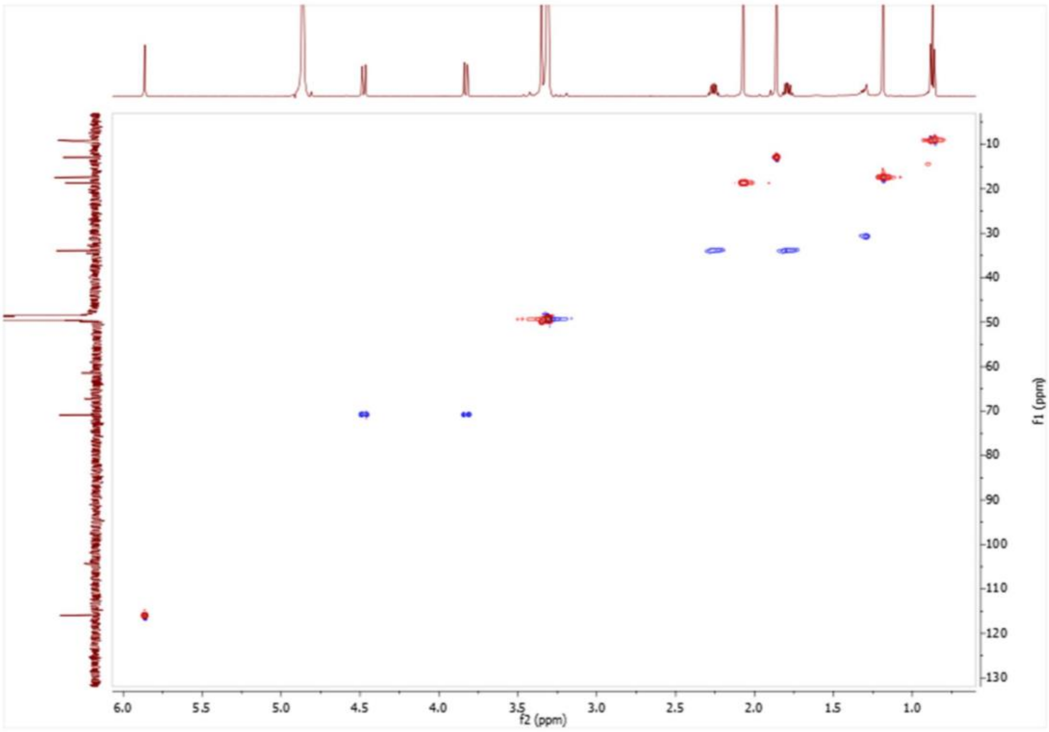

Figure S30. HMBC spectrum of compound **4** (CD_3OD, 600 MHz).

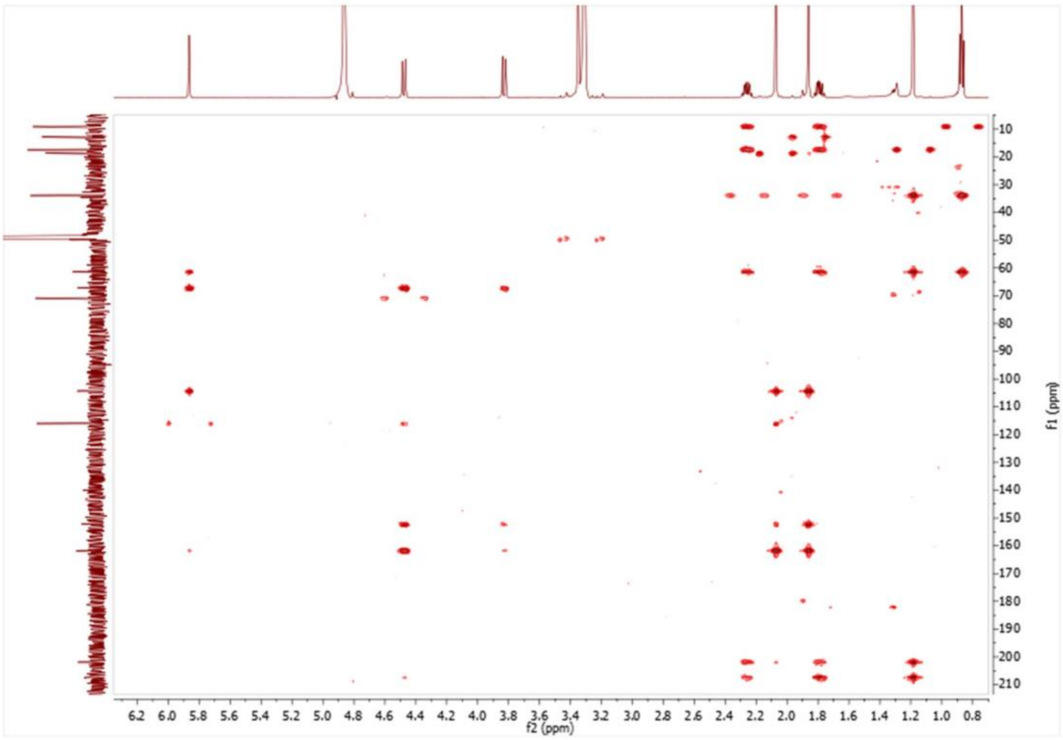

Figure S31. NOESY spectrum of compound **4** (CD_3OD, 600 MHz).

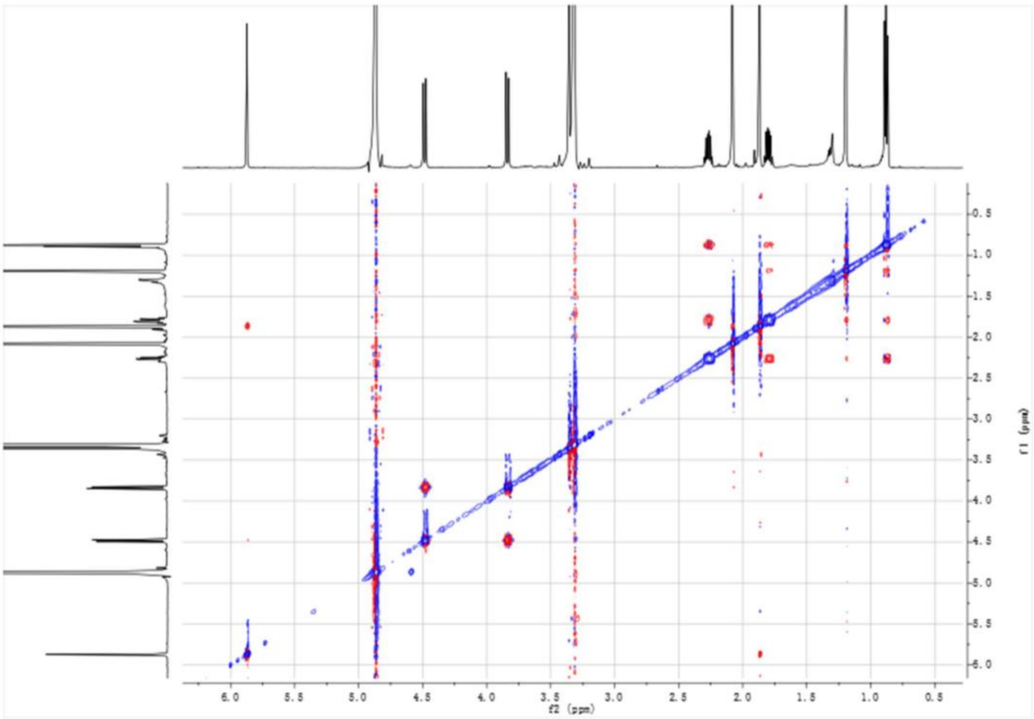

Figure S32. ^{1}H NMR spectrum of compound **4** ($CDCl_3$, 600 MHz).

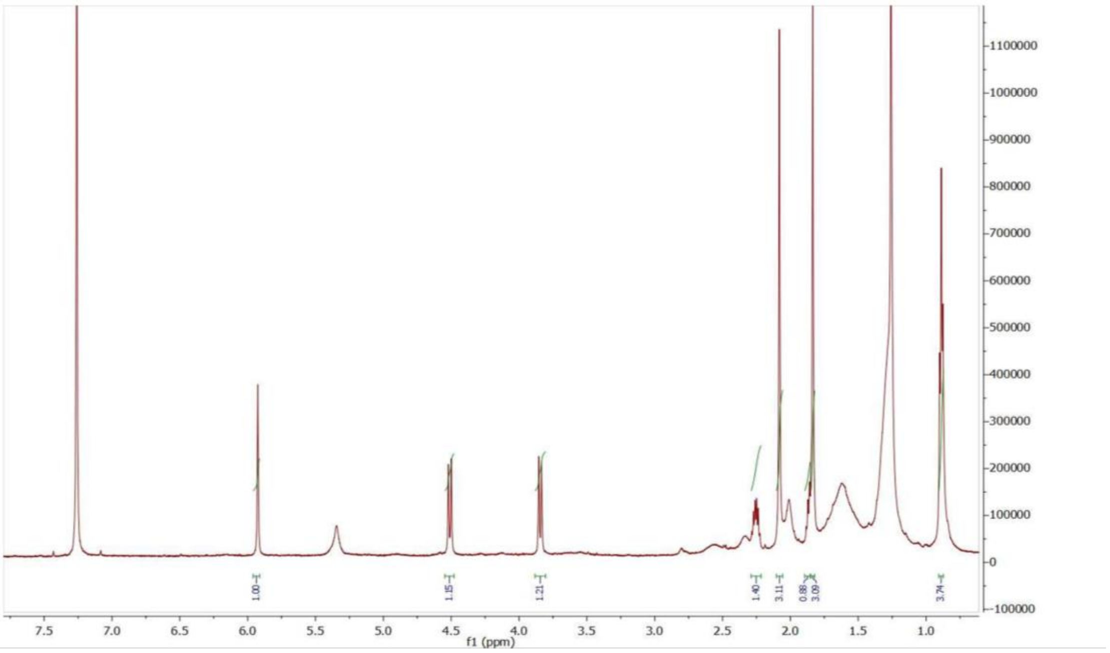

Figure S33. HR-ESIMS spectrum of compound **4.**

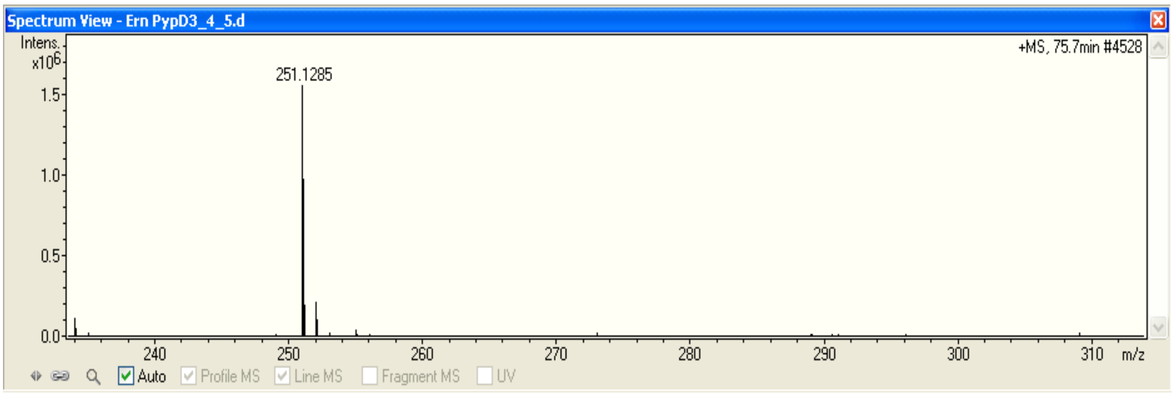

Figure S34. FT-IR spectrum of compound **4.**

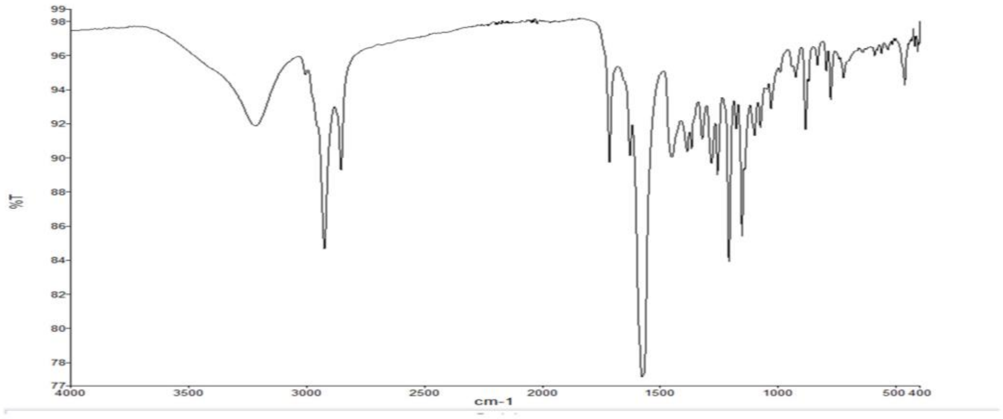

Figure S35. ^{1}H NMR spectrum of compound **5** ($CDCl_3$, 600 MHz).

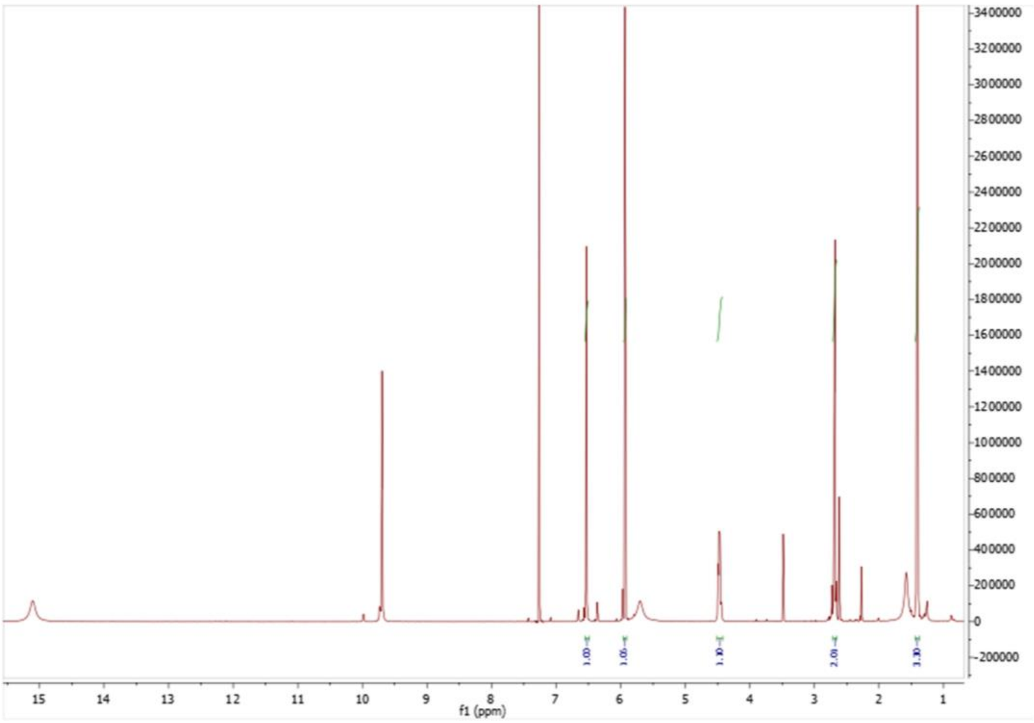

Figure S36. ^{13}C NMR spectrum of compound **5** ($CDCl_3$, 150 MHz).

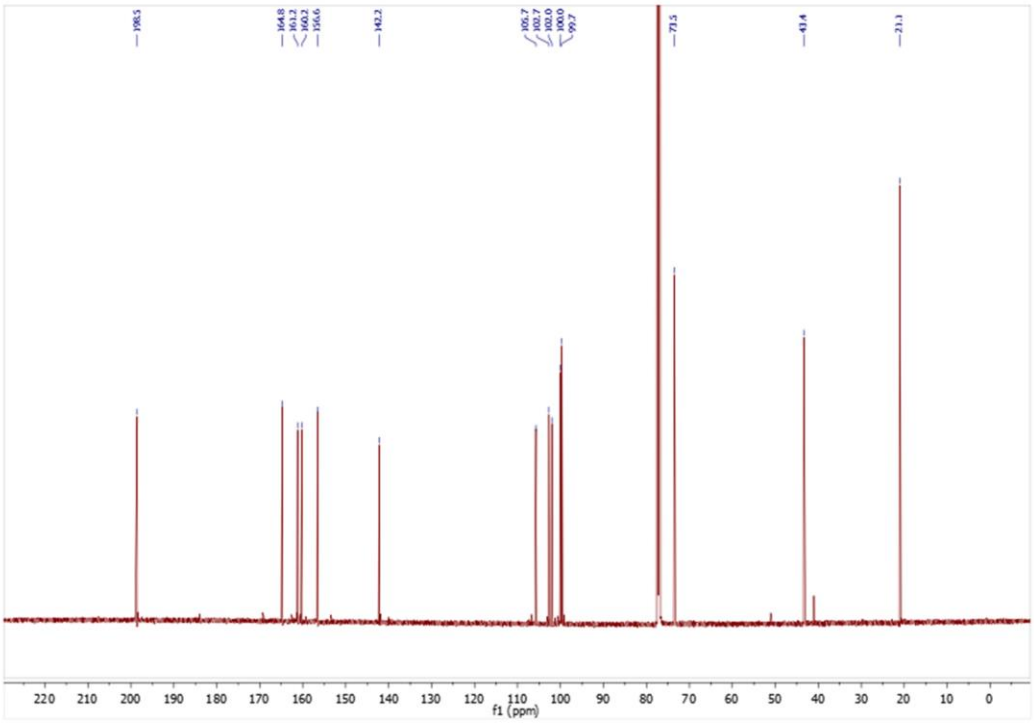

Figure S37. DEPT-135 spectrum of compound **5** ($CDCl_3$, 150 MHz).

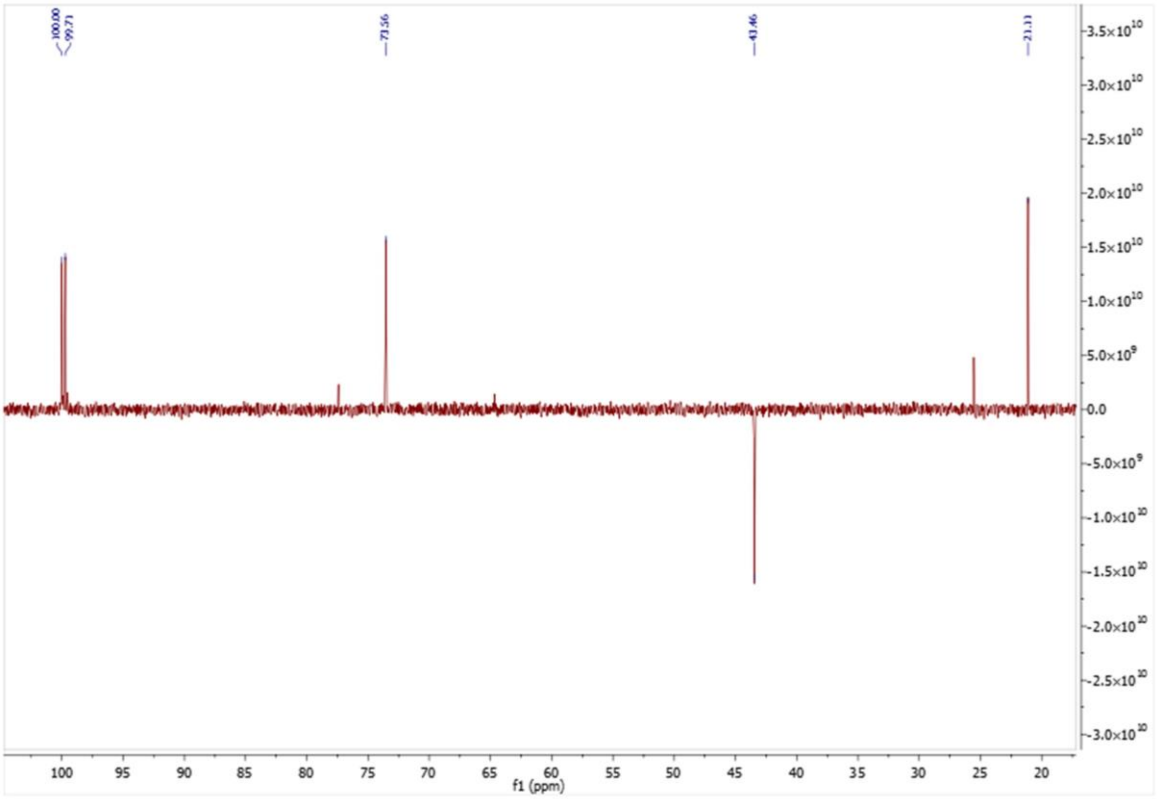

Figure S38. HR-ESIMS spectrum of compound **5.**

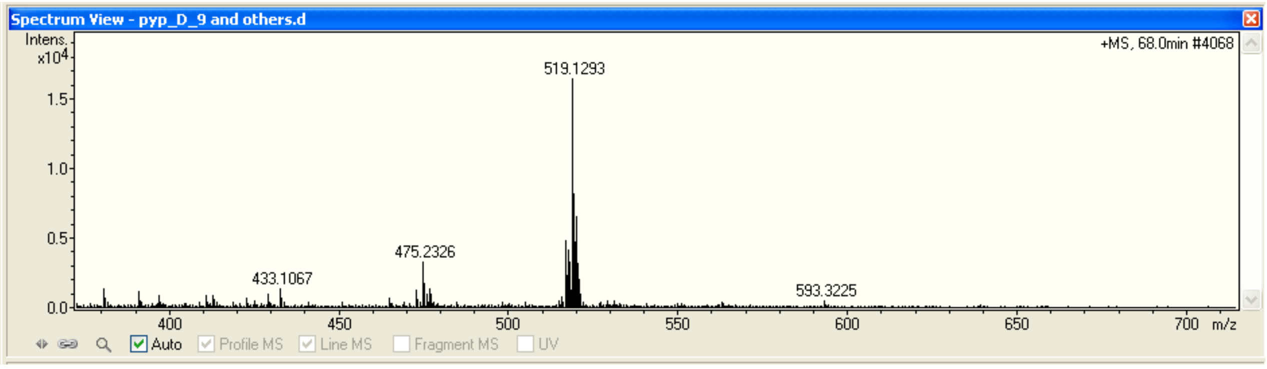

Supplement: Supplementary file 1 [file marinedrugs-18-00073-s001.pdf]
